# Isotomidae of Japan and the Asiatic part of Russia. I. *Folsomia ‘inoculata*’ group

**DOI:** 10.3897/zookeys.750.22764

**Published:** 2018-04-16

**Authors:** Mikhail Potapov, Motohiro Hasegawa, Natalia Kuznetsova, Anatoly Babenko, Alexander Kuprin

**Affiliations:** 1 Moscow State Pedagogical University, Kibalchich str., 6, korp. 3, Moscow 129278, Russia; 2 Shikoku Research Center, Forestry and Forest Products Research Institute, Kochi 780-8077, Japan; 3 The Severtsov Institute of Ecology & Evolution, Russian Academy of Sciences, Leninski pr. 33, Moscow 119071, Russia; 4 Federal Scientific Center of the East Asia Terrestrial Biodiversity, Far Eastern Branch of the Russian Academy of Sciences, Vladivostok-22, 690022, Russia

**Keywords:** α-taxonomy, Collembola, Far East of Russia, Japan, key

## Abstract

The paper considers blind species of the genus *Folsomia* having two pairs of macrosetae on both meso- and metathorax and united in so-called ‘*inoculata*’ group, which is given a new, more laconic definition. Morphological characters important in the group’s taxonomy are discussed and a further division into four subgroups is proposed. Eight new species, i.e., *F. amurica* Potapov & Kuznetsova, **sp. n.**, *F. breviseta* Potapov & Kuznetsova, **sp. n.**, *F. calcarea* Potapov, **sp. n.**, *F. imparis* Potapov & Hasegawa, **sp. n.**, *F. laconica* Potapov & Kuznetsova, **sp. n.**, *F. tertia* Potapov, **sp. n.**, *F. trisensilla* Potapov, **sp. n.**, and *F. tubulata* Potapov & Babenko, **sp. n.**, are described. *F. hidakana* Uchida & Tamura and *F. inoculata* Stach are redescribed basing on new material, for the latter species the Stach’s individuals were also examined. A key to species of the group is given.

## Introduction

The present revision is based on a vast material recently collected by the authors in various parts of the Eastern Palaearctic and older collections kindly provided by our colleagues. All used materials are deposited in the Tottori Prefectural Museum (Japan) and Moscow State Pedagogical University (Russia).

Traditional methods of morphological taxonomy were mainly used. Multi-dimensional scaling was also applied for variability analysis of widespread *F. inoculata*. Nine metric characters (ratios) were defined for 84 individuals from five large regions of the Palearctic (see the legend to Fig. 89). Metric characters were inferred from body measurements as following ratios: PAO length : inner edge of unguis, PAO length : width of Ant.I, manubrium length: mucro length, dens length: mucro length, dens length: macrosetae length at the end of abdomen, head diagonal : PAO length, macrosetae length at the end of abdomen : mucro length, accp2-s length : accp3-s length (Abd.V), macrosetae length at the end of abdomen : accp3-s length (Abd.V). The correlation index was used to estimate the distance between individuals. Only adults and subadults of a similar size were studied for this analysis, as well as for the species descriptions.

**Abbreviations used**:

**Abd.** abdominal segments;

**alt.** altitude;

**Ant.** antennal segments;

**AO** antennal organ;

***bms*** basal *ms* on antennal segments;

***Md, Mdl, Ml*** macrosetae in dorsal, dorso-lateral and lateral position;

***ms*** micro *s*-seta(e) or *ms*-setae;

**MSPU** Moscow State Pedagogical University;

**PAO** postantennal organ;

***s*** in the text and figures – macro *s*-setae or *s*-setae;

**Th.** thoracic segments;

**TPM** Tottori Prefectural Museum

## Taxonomy

### Remarks on *Folsomia inoculata* group

The group was firstly characterized by three basic characters, i.e., the presence of dorsal macrosetae on Th.II-III, posterior position of medial s-setae on abdominal segments, and the presence of ventral setae on Th.III (Potapov 2001).

Now it became clear that there are some important exceptions, namely three species with mid-tergal position of s-setae (*breviseta* sp. n., *calcarea* sp. n., and *torpeda*) and two species without ventral setae on Th.III (*breviseta* sp. n. and *hidakana*). Otherwise, all these species are obvious members of the same very characteristic East Asiatic group. This fact forces us to propose a new definition of the group: *Folsomia* with eyes absent, macrosetae on dorsum (= Md) of each Th. II and III present resulting in 22/333 formula, body shape tubular, head massive, PAO long and slender.

All species of the group also share several ordinary characters: four sublobal hairs on maxillary outer lobe, bifurcate maxillary palp, labral formula 4/5,5,4, not reduced edge of labrum, unguis without lateral or inner teeth, the absence of foil setae at the tip of abdomen. Therefore, we exclude all these characters from the species diagnoses given below.

### Classification of the group

The group is not homogenous and its members can be classified by appearance into three types: ‘long-furcated’ common for *Folsomia*, ‘short-furcated’ slender cylindrical (‘*tatarica*’ subgroup), and stout with massive head (*F. inoculata*). Moreover, the group can be divided into four subgroups basing on well visible although not necessary evolutionary significant characters:

– ‘*hidakana*’ subgroup. It consists of the most primitive species having long furca, complete sets of s-setae and common (for the genus) number of ms-setae on body (43/22235, 10/100, as in Figs 21, 22) and without ventral setae on Th.III (Fig. 10). *F. breviseta* sp. n., *F. hidakana*.

– ‘*macrochaetosa*’ subgroup. Unlike the previous group, its species have ventral setae on Th.III (Fig. 9). *F. amurica* sp. n., *F. brevisensilla*, *F. imparis* sp. n., *F. macrochaetosa*, *F. setifrontalis*. An odd species, *F. bashkira*, characterized by unusual reduction of s-setae at the middle part of the body (42/11235), in other features resembles these species and so is also placed into this subgroup.

– ‘*laconica*’ subgroup. Species with incomplete set of s-setae (33/22224) and ms-setae (10/000) on body (as in Figs 55, 56). *F. laconica* sp. n., *F. tertia* sp. n., *F. trisensilla* sp. n. Other characters as in ‘*macrochaetosa*’ subgroup.

– ‘*tatarica*’ subgroup. The species are habitually specific due to slender body (Figs 69, 70), rather short macrosetae and short furca. They have complete set of s-setae (43/22235), ms of Abd.I present or absent. *F. baida*, *F. calcarea* sp. n., *F. tatarica*, *F. torpeda*, *F. tubulata* sp. n.

*Folsomia inoculata* holds a unique position in the group due to s-pattern on Abd.V, chaetotaxy of furca, and specific appearance (for details see Remarks for the species).

### The state of knowledge of the group

We believe that representatives of the ‘*inoculata*’ group were previously collected and recorded by other researchers in the eastern areas of Asia. In the associated regional papers (Yosii 1939, 1977; Tanaka 1970, Lee 1973; Kurcheva 1977; Solntseva and Molodova 1979; Suma 1997; Yamauchi and Suma 1999; Furuno et al. 2000; Niijima and Hasegawa 2011; Hishi et al. 2012) they were possibly listed as either ‘*F. fimetaria*’, ‘F. cf. fimetaria’ or ‘*Folsomia* sp.’ Indeed, species of the ‘*inoculata*‘ group share several superficial characters with ‘*fimetaria*’ group members and so could be confused with them. If considering essential characters, PAO in the latter group is oval and short, thoracic Md macrosetae are absent, and abdominal tip often has foil setae.

### Distribution and ecology

The known species of the group mostly inhabit the boreal zone of the Eastern Asia. If considering our unpublished materials from North America, one species (*F. inoculata*, together with its junior synonym *F. ezoensis*) has almost trans-Holarctic range. Subgroup of short-furcated species (‘*tatarica*’ sgr.) occupies areas close to the Ural Mts (excl. *F. tubulata* sp. n.) although does not penetrate to the main part of Europe. In North America (unpubl. material, coll. A. Fjellberg) the group is not so diverse and we have discovered only few, mostly new species. Representatives of the ‘*inoculata*’ group often predominate in litter of native forests and such ecological niche may be used as an additional difference from the ‘*fimetaria*’ group. The latter group prefers various disturbed habitats, organically enriched sites, etc. (*F. candida*, *F. fimetaria*, and *F. litsteri*, for example).

### Key taxonomic characters of the group

**Figures 1–8:**
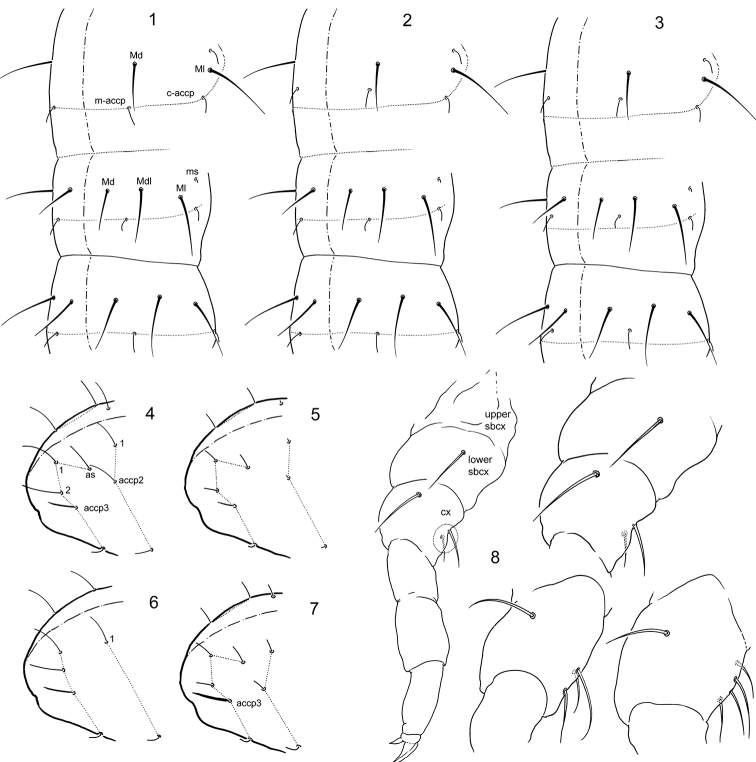
. *Folsomia* spp. **1–3** Variants of position of medial *s*-setae on Th.III, Abd.I, and Abd.II **4–7** Sensillar patterns at the end of abdomen in *F. amurica* sp. n. (**4**), *F. bashkira* (**5**), *F. laconica* sp. n. (**6**), and *F. inoculata* (**7**) **8** Position and different number (two, three, and four) of frontal setae on coxa of leg I (encircled). Abbreviations: Md, Mdl, Ml–dorsal, dorso-lateral and lateral macroseta, m-accp, c-accp–medial and corner accp-s-setae, ms–ms-seta, as–as-s-seta, accp2, accp2 –accp-s-seta, upper sbcx, lower sbcx–upper and lower subcoxae, cx–coxa.

*Position of s-setae on tergites.* The group show a high diversity of position of medial s-setae relatively to p-row – four patterns can be discriminated (Table 1). Three most frequent variants are shown in Figs 1–3.

**Table 1.** Position of medial s-setae on tergites in the ‘*inoculata*’ group.

Th.II–III	Abd.I–II	Abd.III	Species
in p-row	in p-row	in p-row	*F. amurica* sp. n., *F. imparis* sp. n., *F. laconica* sp. n., *F. macrochaetosa*, *F. setifrontalis*, *F. tertia* sp. n., *F. trisensilla* sp. n., *F. baida*, *F. tubulata* sp. n.
in front of p-row	in p-row	in p-row	*F. bashkira*, *F. brevisensilla*, *F. hidakana*, *F. inoculata*
in p-row	in p-row	in front of p-row	*F. tatarica*
in front of p-row	in front of p-row	in front of p-row	*F. breviseta* sp. n., *F. calcarea* sp. n., *F. torpeda*

*S-pattern on Abd.IV and V.* Position and differentiation of s-setae on Abd.V is one of the keys to understanding of evolution of the genus *Folsomia* (Potapov and Greenslade 2010). Most species of the ‘*inoculata*’ group show ‘4+1’ or weakly differentiated ‘3+1+1’ s-pattern widely distributed in the genus (Fig. 4). Three new species (*F. laconica* sp. n., *F. tertia* sp. n., and *F. trisensilla* sp. n.) loose two s-setae: lateral accp-s in dorsal pair of s-setae on Abd.IV and anterior as-s on Abd.V resulting in a pattern showing in Fig. 6. These three species also loose corner accp-s on Th.II and ms on Abd.I (Figs 55, 58, 65). These three s-setae loss (on Th.II, Abd.IV, and Abd.V) is a unique character for the genus: s-formula 33/22224 (instead of common for the genus 43/22235) was known neither in the ‘*inoculata*’ group nor in the genus, although similar but not identical reduction is typical of several species of the ‘*sensibilis*’ group (33/22225). In the latter case, as-s-setae of Abd.V remain in the set. Another trend is a differentiation of complete terminal set: s-setae of Abd.IV can undergo shortening (Fig. 5). Differentiation of Abd IV–V s-pattern is most marked in *F. inoculata* (Fig. 7).

*Front setae on coxa of leg I.* Species of the ‘*tatarica*’ subgroup, *F. inoculata*, *F. brevisensilla*, *F. breviseta* sp. n., and *F. tertia* sp. n. have two such setae, *F. laconica* sp. n., *F. trisensilla* sp. n., and species of the ‘*macrochaetosa*’ subgroup – three, and *F. hidakana* – four (Fig. 8). This trait probably reflects general number of setae on legs and furca while shows some exceptions.

**Figures 9–14.**
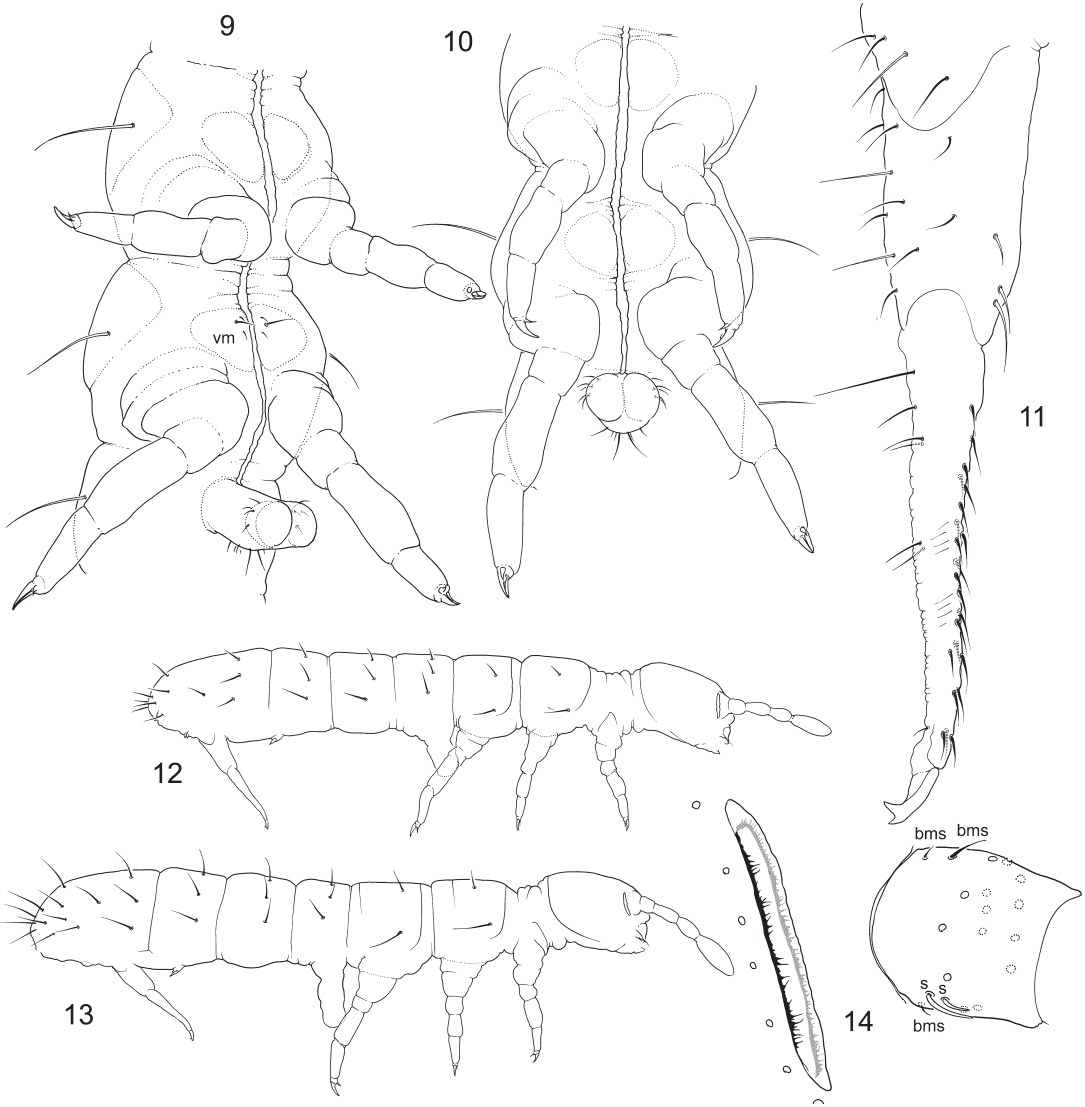
*Folsomia* spp. **9–10** Ventrum of Th.II, Th.III, and Abd.I in *F. imparis* sp. n. (**9**), setae on Th.III present and *F. hidakana* (**10**), setae absent **11–12, 14** *F. breviseta* sp. n., furca, lateral view (**11**), appearance (**12**), PAO and Ant.I (**14**) **13** Appearance of *F. hidakana*. Abbreviation: vm–ventro-medial setae.

*Setae on ventrum of metathorax.* The species of the ‘*inoculata*’ group normally have three ventral setae on each side of Th.III, one of which is long and two are short (Fig. 9). Several species have fewer or more variable number of ventral thoracic setae or all these setae are subequal in size (*F. inoculata*, *F. brevisensilla*, and all species of the ‘*tatarica*’ subgroup). Two species sharply differ from other congeners of the group having no ventral setae on Th.III (*F. breviseta* sp. n., *F. hidakana*) (Fig. 10).

### Characters of lower taxonomic value

– PAO in all ‘*inoculata*’ species is slender, with parallel edges, and more than 1.3 times longer than width of Ant.I (Figs 14, 24, 62, 78) that is probably a sharp characteristic of the group discriminating it from adjoining ‘*fimetaria*’ and ‘*sensibilis*’ groups. The so-called “inner denticles” along its edges and middle constriction are usually seen in all species in different extend while both characters considerably vary within populations.

– All species have three basal ms-setae on Ant.I: one ventral and two dorsal. Two dorsal bms-setae are arranged in a longitudinal line, proximal bms is longer (Figs 14, 23, 24). The proximal bms usually hardly differs from common setae and herewith should be carefully excluded if calculating common setae on Ant.I. Species of the ‘*tatarica*’ subgroup have proximal bms clearly shorter than common setae (Fig. 78) while its length also varies depending on specimens.

– The members of ‘*hidakana*’, ‘*macrochaetosa*’, and ‘*laconica*’ subgroups have minute subapical setae on posterior side of dens. The size of the seta varies depending on specimens and often hardly detectable. Small wrinkle in which this seta set in is always visible.

### Descriptions of species

#### Subgroup ‘*hidakana*’

##### Folsomia breviseta

Taxon classificationAnimaliaCollembolaIsotomidae

Potapov & Kuznetsova sp. n.

http://zoobank.org/62BCDE0A-6F11-4162-B7EA-58A0B2061542

Figs 11 , 12 , 14 , 15–20

###### Type material.

Holotype, female, Russia, NE Yakutia, Middle Indigirka, near Ust-Nera, 700 m alt., larch forest with thick lichen cover, litter, 22.vii.1992. Ten paratypes from the same biotope, five paratypes from mountain tundra (1500 m alt.) with bushes of *Betula nana* and one paratype from mosses in stony niche on northern slope (1600 m alt.) in the same location. All collected by N. Kuznetsova and M. Potapov. Deposited in MSPU.

###### Diagnosis.

Blind. Dorsal macrosetae (Md) present on both Th.II and Th.III. Sensillary formula complete (43/22235; 10/100). Medial s-setae on body tergites short, set in anterior position. Macrosetae short. Ventral setae on Th.III absent. Anterior side of manubrium normally with 2+2 setae, dens with 18–22 anterior setae. Mucro bidentate.

###### Description.

**Figures 15–20.**
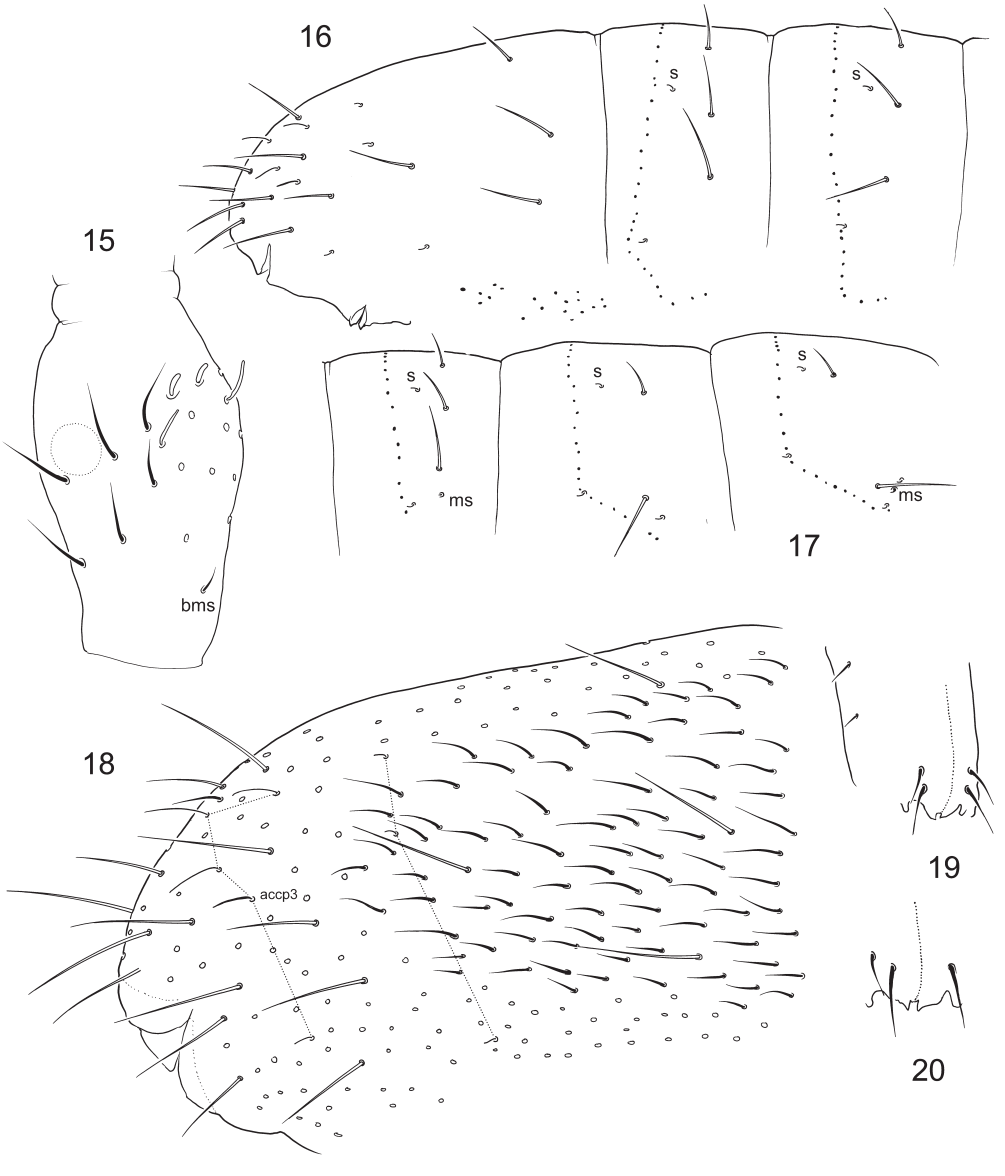
*F. breviseta* sp. n. **15** Ant.III (area of common position of lateral s-setae marked) **16–17** Position of macrosetae, setae of p-row, and s-setae on posterior (**16**) and anterior (**17**) half of corpus **18** Chaetotaxy of Abd. IV–VI **19–20** Chaetotaxy of anterior side of manubrium, variations. bms–basal ms.

Body size from 1.0 (one adult male) to 1.7 mm. Body shape relatively tubular, general appearance not typical of ‘*inoculata*’ group due to short macrosetae (Fig. 12). Without ocelli and pigmentation. Cuticle with fine, hexagonal primary granulation (“smooth”). PAO slender and long, middle constriction varies, ‘inner denticles’ usually well developed, PAO length 1.8–1.9 as long as width of Ant.I and 2.3–2.5 as long as inner unguis length (Fig. 14). Labium complete, guard setae e7 present, three proximal and four basomedian setae. Ventral side of head with 4+4 postlabial setae. Ant.I with 13–15 common setae, two ventral s-setae (s) and three basal micro s-setae (bms): two dorsal bms (short and long) and one ventral bms. Ant.II with three bms and one latero-distal s, Ant.III with one bms and four distal s (lateral s absent, Fig. 15). Several tubular s-setae on Ant.IV. Organite small.

Common setae short. Sensillary formula as 43/22235 (s) and 10/100 (ms). S-setae short, four s-setae on dorsal side of Abd.V longer. Medial s-setae on Th.II–Abd.III situated in front position, on Abd.I–III between Md and Mdl (Figs 16, 17). Abd.V with five s-setae arranged as three in dorsal position, thin, as long as common setae (as, accp1, accp2), one lateral, thicker than dorsal, and one latero-ventral, short (‘3+1+1’ pattern) (Fig. 18), accp3 s-setae subequal to accp2. Macrosetae smooth and short, 2,2/3,3,3 in number, medial ones on Abd.V much shorter than dens (2.3–3.1) and 1.8–2.3 times longer than mucro (Fig. 18). Axial chaetotaxy as 10–12,7–8,/5–6,5,5. Thorax without ventral setae.

Empodial appendage as long as 0.5–0.6 unguis. Tibiotarsi with 24–27 setae on legs I–II, and 28–32 on leg III. Upper and lower subcoxae of legs I–III with 0,1/3,6–8/5–7,7–9 setae, respectively. Coxae of leg I with two front setae. Ventral tube with 4+4 latero-distal and 5–7 posterior setae (two in distal transversal row and 3–5 in more proximal position), anteriorly without setae. Tenaculum with 4+4 teeth and a seta. Anterior furcal subcoxae with 12–15, posterior one with six setae. Anterior side of manubrium normally with 2+2 setae at distal edge, arranged in two longitudinal lines (Fig. 19). One additional seta often present on one side in a distance from main group resulting in 2+3 set (Fig. 11). One of the paratypes shows 2+1 anterior setae on manubrium (Fig. 20). Posterior side of manubrium with 4+4 latero-basal, two apical setae (ap), 2+2 setae in distal transversal row (M1, L1), two pairs of lateral setae, and 5–6+5–6 in central part (Fig. 11). Dens with 18–22 anterior setae. Posterior side of dens crenulated and with seven setae: four basal, two at the middle, and one (not especially small) at base of mucro (Fig. 11). Mucro bidentate. Ratio of manubrium : dens : mucro = 4.1–4.5 : 5.1–5.8 : 1. Males present.

###### Remarks.

*Folsomia breviseta* sp. n. combines several characters rare for the group: short macrosetae and s-setae, anterior position of medial s-setae on body tergites, the absence of ventral setae on Th.III. A loss of lateral s-setae on Ant.III is a very peculiar character while the nature of this character is not easy to interpret since all species of the family Isotomidae, if not all Collembola, have these s-setae. The absence of s-setae was confirmed in all available specimens. Beyond ‘*inoculata*’ group several blind species with two pairs of setae on manubrium can be confused with *F. breviseta* sp. n., for example, *F. bisetosa* Gisin, *F. cephalota* Bu et al., and *F. sensibilis* Kseneman. All these species belong to other groups and therefore differ essentially by characters of high rank.

###### Distribution and ecology.

The species is known only from the type locality where it inhabits mountain forests and tundra.

###### Derivatio nominis.

The new species name reflects short setae on body.

##### Folsomia hidakana

Taxon classificationAnimaliaCollembolaIsotomidae

Uchida & Tamura, 1968

Figs 10 , 13 , 21–27 , 28 , 90

###### Material.

Japan, Honshu Island, Nobeyama, Nagano, 10.x.2012, coll. M. Hasegawa; Kitaibaraki, 2.xi.2011, coll. M. Hasegawa; Hokkaido Island. Shore of Harutori Lake, Kushiro city, 14.v.2014 and 19.viii.2014, coll. Y. Suma; Shiretoko Peninsula, surroundings of Utoro, litter of mixed forest, 90 m alt., 20.viii.2016, 44.1006°N, 145.0584°E, coll. M. Potapov and N. Kuznetsova; Shiretoko Peninsula, trail to Rausu Mount, ~200 m alt., 29.ix.2013, coll. R. Kitagawa and S. Fujii; ibidem, trail to Rausu Mount, ~1000 m alt, oak litter, coll. M. Potapov and N. Kuznetsova.

North Korea. Hamgyong-namdo Province (= South Hamgyong), valley N from Song-riong, 03.vi.1987, and SW from Tanchon, 30.v.1987, coll. A. Szeptycki.

Far East of Russia, Primorsky Krai, Lazovsky District, nearby Preobrazheniye, litter and rotten wood in deciduous and coniferous forests, 400–700 m alt., 21.ix.2011, coll. M. Potapov, Y. Bu, H. Chen-Wang; Khasansky District, Peschany Peninsula, near Beregovoye, litter under dogrose, 09.ix.2004, coll. M. Potapov, L. Deharveng, R. Pomorski, and A. Bedos, Khasansky District, ~15 km S Kraskino, Mramorny Cape, oak wood litter and soil under coastal reed, 28.ix.2004, coll. M. Potapov, L. Deharveng, R. Pomorski, and A. Bedos; Khasansky District, ~15 km W Kraskino, Mayachnoye (Chertova Gorka), forest litter, 28.ix.2004, coll. M. Potapov, L. Deharveng, R. Pomorski, and A. Bedos; Khasansky District, Krabbe Peninsula, Astafyeva Cape, deciduous litter, v.2007, coll. E. Sokolova; Khasansky District, “Kedrovaya Pad”, mixed forest, litter, 29.vii.2016, coll. M. Potapov and N. Kuznetsova; ibidem, coniferous and deciduos litter, 29.ix.2004, coll. M. Potapov, L. Deharveng, R. Pomorski, and A. Bedos; ibidem, 03.x.2009, coll. O. Smirnova, ibidem, v.2015, coll. A. Matalin; Khasansky District, near Barabash, oak wood on slope, litter, 27.ix.2004, coll. M. Potapov, L. Deharveng, R. Pomorski, and A. Bedos; Partizansky District, vicinities of Ekaterinovka, Chondalaz (= Lazovy) Range, oak litter, 26.ix.2004, coll. M. Potapov, L. Deharveng, R. Pomorski, and A. Bedos; Ussuriyski District, Ussuriyski Reserve, decaying wood, 5.x.2004, coll. M. Potapov, L. Deharveng, R. Pomorski, and A. Bedos; Shkotovsky District, Khualaza Mount, 2.x.2004, coll. R. Pomorski; between Vladivostok and Artem, botanical garden, litter of mixed forest, ix.2012 and 04.x.2009, coll. O. Smirnova; Terneysky District, Sikhote-Alimski Reserve, Kabany station, forest litter, 08.viii.2017, coll. N. Kuznetsova, A. Geras’kina, A. Kuprin; Kavalerovski District, road Kavalerovo-Dal’negorsk, 44.3844°N, 135.3639°E, mossy larch forest with *Rhododendron*, 09.viii.2017, coll. N. Kuznetsova, A. Geras’kina, A. Kuprin; Khabarovsky Krai, Vaninsky District, nearby Datta, coastal larch-wood, 28.ix.2011, coll. M. Potapov; Vaninsky District, five km N Vysokogorny, valley of Mulinka River, larch-forest litter, ~ 600 m alt., 29.ix.2011, coll. M. Potapov.

###### Description.

Body size from 1.2 to 1.7 mm. Body shape as common for the group, not slender (Fig. 13). Usually with large pigment grains rarely scattered on body, more on fused Abd.IV–VI. Cuticle with fine hexagonal primary granulation (“smooth”). Ocelli absent. PAO slender, with clear middle constriction, ‘inner denticles’ hardly developed, its length 1.2–1.7 as long as width of Ant.I and 1.6–2.0 as long as inner unguis length (Fig. 24). Labium complete, guard setae e7 present, three proximal and four basomedian setae. Ventral side of head with 4+4 postlabial setae. Ant.I with 15–17 common setae, 2–3 (see the discussion below, Figs 23, 24) ventral s-setae (s) and three basal micro s-setae (bms): two dorsal bms (short and long) and one ventral bms. Ant.II with three bms and one latero-distal s, Ant.III with one bms and with 5–6 distal s (one or two lateral s, Fig. 25). Organite varies in shape, often large.

**Figures 21–27.**
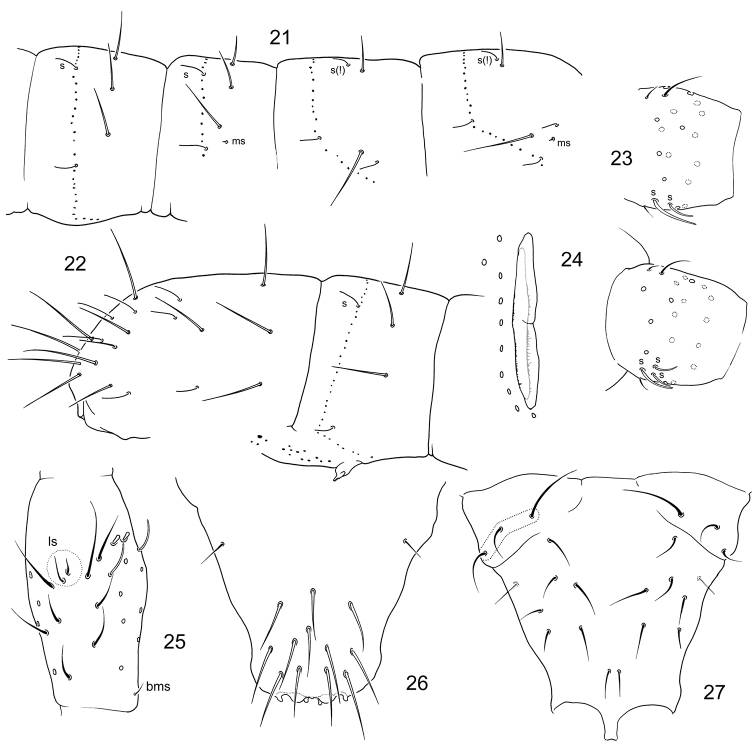
*F. hidakana* **21–22** Position of macrosetae, setae of p-row, and s-setae on anterior (**21**) and posterior (**22**) half of corpus **23** Ant.I (S Primorye: Far East of Russia) **24** PAO and Ant.I (Honshu: Japan) **25** Ant.III **26–27** Manubrium, anterior (26) and posterior (27) views. Abbreviations: bms–basal ms, ls–lateral s.

**Figures 28–35.**
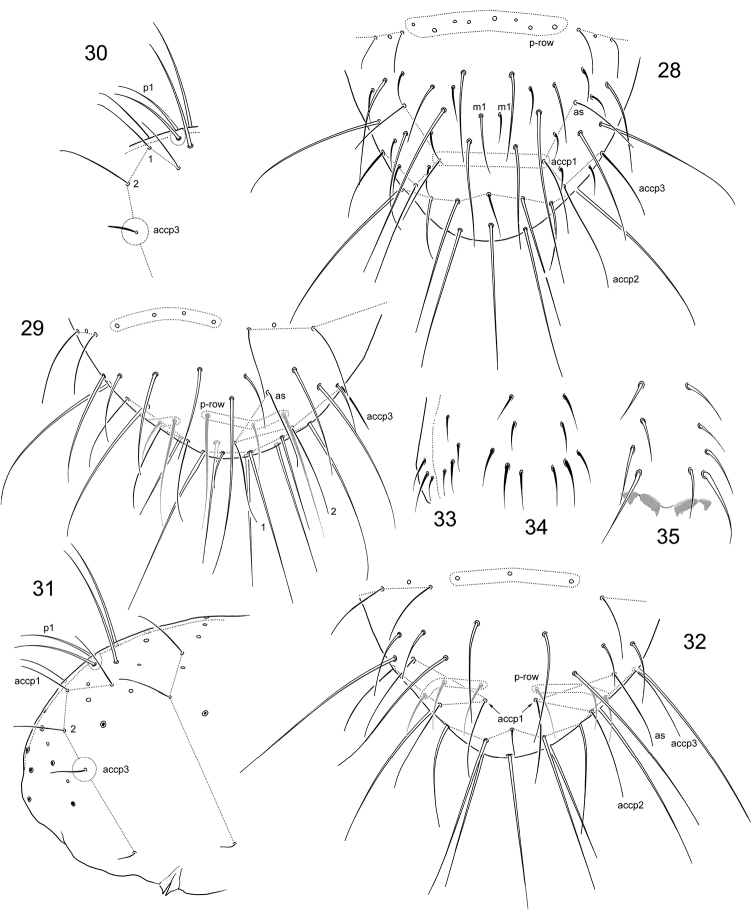
*Folsomia* spp. **28** *F. hidakana*, end of abdomen, dorsal view **29–31** *F. amurica* sp. n., end of abdomen, specimens from Amurskaya Region, dorsal (**29**) and lateral views (**31**), specimen from Inner Mongolia Province, lateral view (**30**) **32** *F. imparis* sp. n., end of abdomen **33–35** *F. amurica* sp. n., chaetotaxy of anterior side of manubrium, variations.

Common setae middle-sized. Setae covering polychaetotic: Abd.IV with 7–9 p-setae between medial accp-s, Abd.V with m1-setae (marked on Fig. 28). Sensillary formula as 43/22235 (s) and 10/100 (ms). S-setae long and thin, as long as common setae. Medial s-setae on Th.II–III situated in front position, nearby Md macrosetae, on Abd.I–III in p-row, between Md and Mdl (Figs 21, 22). Abd.V with five s-setae arranged as four in dorsal position, rather long (as, accp1, accp2, accp3), and one latero-ventral, middle-sized (weakly differentiated ‘4+1’ pattern), accp3 s-setae insignificantly shorter and thicker than accp2 (Fig. 28). Macrosetae smooth, their length vary from long to moderately long, 2,2/3,3,3 in number, medial ones on Abd.V a little shorter than dens (1.0–1.3) and 3.7–6.0 times longer than mucro. Axial chaetotaxy as 9–12,7–9,/4–5,4–5,4. Thorax without ventral setae (Fig. 10).

Empodial appendage approximately half as long as unguis. All tibiotarsi with many additional setae: 29–33 on legs I–II, ~38–42 on leg III. Upper and lower subcoxae of legs I–III with 0,1/4–6,8–11/8–12,8–11 setae, respectively. Coxae of leg I with four (rarely five) front setae. Ventral tube with 5–6+5–6 latero-distal and 6–8 posterior setae, anteriorly without setae. Tenaculum with 4+4 teeth and a seta. Anterior furcal subcoxae with 11–14, posterior one with four setae. Anterior side of manubrium with 4–6+4–6 pair setae and usually with two unpaired axial setae (Fig. 26). Posterior side of manubrium with 3+3 latero-basal, two apical setae (ap), 2+2 setae in distal transversal row (M1, L1), one pair of lateral setae, and 4(3)+4(3) in central part (Fig. 27). Dens with 19–23 anterior setae. Posterior side of dens crenulated and with six setae: three basal, two at the middle, and one rudimentary at base of mucro (often hardly visible). Mucro bidentate. Ratio of manubrium : dens : mucro = 3.7–6.2 : 4.5–7.0 : 1. Males present.

###### Remarks.

Our specimens fit to the original description of *F. hidakana* in all significant features. Uchida and Tamura (1968) did not show in figures a subapical rudimentary seta on posterior side of dens and short latero-central setae (l2) on manubrium (probably overlooked). We also found wider variability in most characters that is certainly explained by larger material we have studied. Macrosetae on figures in first description (Figs 28, 35 in Uchida and Tamura 1968) seem to be shorter than in our material. *Folsomia hidakana* is a peculiar species due to anterior position of medial s-setae on thoracic segments, 5+5 or more latero-distal setae on ventral tube (vs 4+4 that is more common for the group), four setae on posterior furcal subcoxa (fewer than common for the group), 3+3 latero-basal and 1+1 latero-central setae on posterior side of manubrium (fewer than common for the group). The absence of ventral setae on Th.III is the main differentiated feature of *F. hidakana* shared only with allopatric *F. breviseta*, the two species are combined in the formal subgroup ‘*hidakana*’ by us. From ‘*macrochaetosa*’ group *F. hidakana* differ by more setae on body and more lateral position of accp1-s on Abd.V (see Fig. 28 vs Figs 29, 32). Being often mixed with habitually similar species (often with *F. imparis* sp. n.), *F. hidakana* is normally easy to recognize by scattered pigment grains on body.

Specimens collected in Japan, both in Honshu and Hokkaido, differ from specimens from Russia by three (vs two) s-setae on Ant.I. In Japanese populations the individuals with two s rarely occur so we keep both variants within diagnosis of *F. hidakana*.

###### Distribution and ecology.

Species was described from Hokkaido (Hidaka-Mombetsu) and subsequently listed in catalogues of Japanese Collembola (Yosii 1977; Furuno et al. 2000; Niijima and Hasegawa 2011). Known from Hokkaido (Suma 1997, 2008, Hishi et al. 2012), eastern Honshu (Ibaraki) (Hasegawa et al. 2009), Aomori Pref. (Yamauchi and Suma 1999, 2009). In Far East of Russia the species was previously recorded near Ussuriysk (Kutyreva 1988) and in Shikotan Island (as ‘F. sp.aff. hidakana’ in Potapov and Marusik 2000).

As a whole, distributional range of the species appears to cover southern area of the Russian Far East, North Korea, and northern half of Japan (Fig. 90). It inhabits forest litter and decaying wood in low mountains, rare in higher altitudes.

#### Subgroup ‘*macrochaetosa*’

##### Folsomia amurica

Taxon classificationAnimaliaCollembolaIsotomidae

Potapov & Kuznetsova sp. n.

http://zoobank.org/3F726CE9-BE32-46F1-88E9-BC0FBED96F0E

Figs 4 , 29–31 , 33–35 , 36–39 , 90

###### Type material.

Holotype, female, Far East of Russia, Amurskaya Region, Zeysky Reserve, ~50 km W Zeya, near “Gol’tsy” station, subalpine dwarf wood (*Pinus pumila*), ~ 1300 m alt., coniferous litter, 20.viii.2014., Ten paratypes from the same biotope and five paratypes from the same location, litter of mixed forest at 700 m alt., 21.viii.2014, coll. M. Potapov and N. Kuznetsova. Deposited in MSPU.

###### Other material.

Far East of Russia, Amurskaya Region, various biotopes nearby type locality: litter and rotten wood in mountain tundra, spruce and larch forests at different altitudes (from 400 to 1400 m alt.); Amurskaya Region, ~ three km W Arkhara, oak-forest, litter, 17.viii.2014, coll. M. Potapov and N. Kuznetsova.

China, Inner Mongolia Province, Da Hinngan Ling Mts, ~ 25 km W BaLin, young tussocky wet birch forest, litter, 12.viii.2014, coll. M. Potapov.

###### Diagnosis.

Blind. Dorsal macrosetae (Md) present on both Th.II and Th.III. Sensillary formula complete (43/22235; 10/100). Medial s-setae on body tergites long, set in p-row. Ventral setae on Th.III present. Manubrium on anterior side with 4–6+4–6 setae, no unpaired axial setae, dens with 23–27 anterior setae. Mucro bidentate.

###### Description.

Body size from 1.0 to 1.5 mm. Body without pigmentation, relatively tubular (Fig. 39). Cuticle with fine, hexagonal primary granulation (“smooth”). Ocelli absent. PAO slender, constricted, 1.3–1.7 as long as width of Ant.I and 1.6–2.0 as long as inner unguis length. Labium with five usual papillae (*A–E*), guard setae e7 absent, three proximal and four basomedian setae. Ventral side of a head with 4+4 postlabial setae. Ant.I with 15 common setae as a rule, two (rarely three) ventral s-setae (s) and three bms, two of which small, dorsal and ventral, the former set together with long seta-form third bms, Ant.II with three bms and one latero-distal s, Ant.III with one bms and with five distal s (including one lateral), without additional s-setae. Several tubular s-setae on Ant.IV. Organite large, rounded.

**Figures 36–39.**
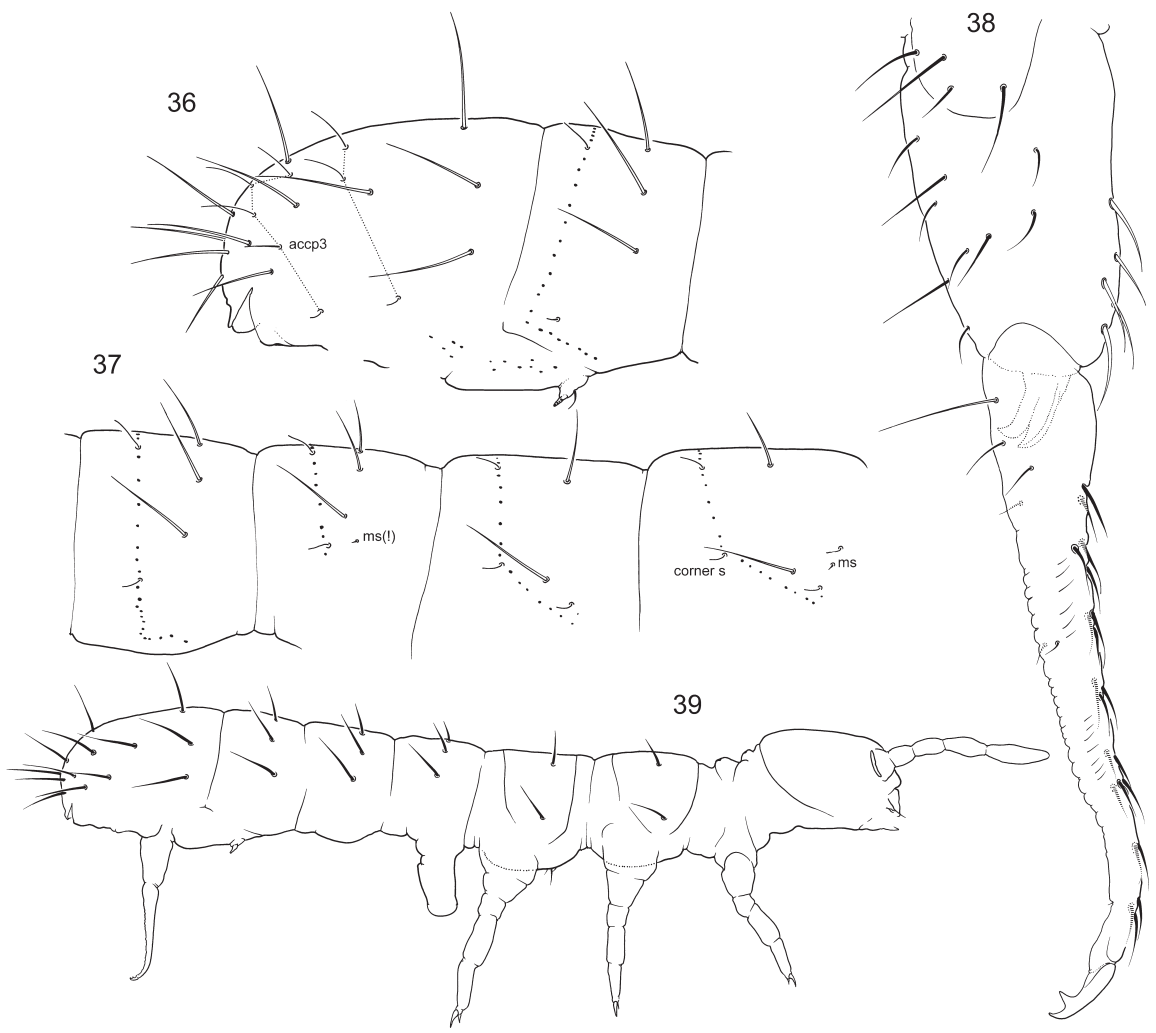
*F. amurica* sp. n. **36–37** Position of macrosetae, setae of p-row, and s-setae on posterior (**36**) and anterior (**37**) half of corpus **38** Furca, lateral view **39** Appearance.

Common setae long. Sensillary formula as 43/22235 (s), 10/100 (ms) (Figs 36, 37). The most tergal s-setae thin and long. Medial s-setae on Th.II–Abd.III situated in posterior position, on Abd.I–III between Md and Mdl. Latero-ventral s-setae on abdominal tergites shorter than medial (Fig. 37). Abd.V with five s-setae arranged as three dorsal ones (as, accp1, accp2), long and slender, one lateral (accp3), clearly shorter (accp2 : accp3 = 1.8–2.3), and one latero-ventral, short (‘3+1+1’ pattern) (Figs 29–31). p1-setae on Abd.V long (see Table 2). Macrosetae smooth and long, 2,2/3,3,3 in number, medial ones on Abd.V 1.5–1.8 times shorter than dens and 4.7–6.2 times longer than mucro. Axial chaetotaxy as 10–11,6,/3–4,3–4,3–4. Metathorax with 3+3 ventral setae of which one long and two short.

Unguis of normal shape, without lateral and inner teeth. Empodial appendage as long as 0.5–0.6 unguis. All tibiotarsi with additional setae: 26–29 on legs I–II and > 35 on leg III. Upper and lower subcoxae of legs I–III with 0,1/3,8–9/5–6,8–10 setae, respectively. Coxae of leg I with three front setae. Tibiotarsal tenent setae pointed, some setae on distal half of tibiotarsi thickened. Ventral tube with 4+4 latero-distal and 7–8 posterior setae (four in distal transversal row and 3–4 in more proximal position), anteriorly without setae. Tenaculum with 4+4 teeth and a seta. Anterior furcal subcoxae with 8–9, posterior one with five setae. Anterior side of manubrium with 4–6+4–6 setae, their position vary, unpaired setae absent (Figs 33–35). Posterior side of manubrium with 4+4 latero-basal, two apical setae (ap), 3+3 setae in distal transversal row (M1, ml1, L1), two pairs of lateral setae, and 3–4+3–4 in central part (Fig. 38). Dens with 19–24 anterior setae. Posterior side of dens crenulated and with six normal setae (four basal and two at the middle) and usually one rudimentary minute seta at the base of mucro (Fig. 38). Mucro bidentate. Ratio of manubrium : dens : mucro = 4.5–6.2 : 7.2–9.3 : 1. Males present.

###### Remarks.

The species most resembles *F. macrochaetosa* and *F. imparis* sp. n. and is characterized by the absence of unpaired setae on anterior side of manubrium, short accp3-s-setae and long p1 setae on Abd.V (Table 2).

###### Distribution and ecology.

Known from three neighboring localities of inner part of East Asia (Fig. 90). The species occurs in forest litter at different altitudes.

###### Derivatio nominis.

The species is common in areas around Amur River lowlands.

##### Folsomia brevisensilla

Taxon classificationAnimaliaCollembolaIsotomidae

Potapov & Babenko, 2000

###### Material.

Far East of Russia, Magadanskaya Region, Ten’kinsky District, village Kulu, 04.ix.1995, coll. S. Bukhkalo.

###### Remarks.

The species resembles *F. inoculata* sharing with the latter species a middle-sized furca, short s-setae on body, their position on tergites, and undifferentiated ventral setae on Th.III. Nevertheless, *F. brevisensilla* does not possess several unique characteristics of the latter species, e.g., large and tubular accp3-s on Abd.V and the absence of subapical seta on posterior side of dens.

###### Distribution.

It is the most northern species of the ‘*inoculata*’ group since known so far only in the basin of Kolyma River (NE Asiatic part of Russia).

##### Folsomia imparis

Taxon classificationAnimaliaCollembolaIsotomidae

Potapov & Hasegawa sp. n.

http://zoobank.org/695BD340-E77E-43FF-B561-05E42B1FE6B8

Figs 32 , 40–46 , 90

###### Type material.

Holotype, female, Japan, Hokkaido Island, Shiretoko Peninsula, trail to Mont. Rausu, deciduous forest (*Acer*, *Quercus*, *Sorbus*, *Magnolia*, *Daphniphillum macropodium*), litter, 19.viii.2016, 354 m alt., 44.1083°N, 145.0893°E, coll. M. Potapov and N. Kuznetsova. Paratypes, 12 specimens from the same location; three specimens from Russia, Far East, S Primorye, Ussuriysky Reserve, Komarovskoye Forest District, Turova Nipple, Khripunovsky Pass, mixed forest with *Pinus koraiensis* on slope, rotten wood, 22.vii.2016, coll. M. Potapov and N. Kuznetsova; four specimens from same region, but Shkotovsky District, trail to Mont. Khualaza, deciduous forest, rotten wood and litter, 21.vii.2016, coll. M. Potapov and N. Kuznetsova. The material from Japan and Russia is deposited in TPM and MSPU, respectively.

###### Other material.

Far East of Russia, Primorsky Krai, Bikin River near confluence to Amba River, mixed forest litter, 29.ix.2009, coll. O. Smirnova; Ussuriyski District, Ussuriyski Reserve, decaying wood, 5.x.2004, coll. M. Potapov, L. Deharveng, R. Pomorski, and A. Bedos; Shkotovsky District, trail to Khualaza Mount, rotten wood, 21.vii.2016, coll. M. Potapov and N. Kuznetsova; Partizansky District, vicinities of Ekaterinovka, Chondalaz (= Lazovy) Range, oak litter, 26.ix.2004, coll. M. Potapov, L. Deharveng, R. Pomorski, and A. Bedos; Khasansky District, vicinities of Barabash, oak litter, 200–400 m alt., 27.ix.2004, coll. M. Potapov; Sakhalin, Kholmsky District, South Kamysh Ridge, Spamberg Mt., moss in mixed and coniferous forests, 14–15.vi.2017, coll. A. Kuprin; Korsakovsky District, vicinities of Korsakov, forest litter, 16.vi.2017, coll. A. Kuprin; Khabarovsky Krai, suburbs of Khabarovsk, Voronez highlands, leaf litter near river bank. 26.iv.2010, coll. M.Potapov; Verkhnebureinsky District, western part of Badjal Range, upper flow of Irungda River (tributory of Amgun’ River), 1900 m alt., subalpine litter and moss, 23.vi.2014, coll. A. Brinev.

North Korea. Hamgyong-namdo Province (= South Hamgyong), SW from Tanchon, 30.v.1987, coll. A. Szeptycki; Yanggang-do Province, Rimjong-su Waterfall, litter, 7.vii.1985, coll. A. Szeptycki.

Material of F. sp. aff. imparis. Khabarovsky Krai (western part), Bureyskoye Reservoir, Nizny Mel’gin Bay, 50.5539°N, 131.3970°E, 12.ix.2009, coll. M. Babykina; Amurskaya Region, ~3 km N Zeya, oak litter, 22.viii.2014, coll. M. Potapov and N. Kuznetsova.

###### Diagnosis.

Blind. Dorsal macrosetae (Md) present on both Th.II and Th.III. Sensillary formula complete (43/22235; 10/100). Medial s-setae on body tergites long, set in p-row. Ventral setae on Th.III present. Manubrium on anterior side with 3–5+3–5 paired and 2–3 unpaired axial setae, dens with 23–27 anterior setae. Mucro bidentate.

###### Description.

Body size from 0.9 to 1.4 mm. Body without pigmentation, its shape as in *F. amurica* sp. n. Cuticle with fine hexagonal primary granulation (“smooth”). Ocelli absent. PAO slender, constricted, 1.4–1.7 as long as width of Ant.I and 1.7–1.9 as long as inner unguis length. Labium with five usual papillae (*A–E*), guard setae e7 absent, three proximal and four basomedian setae. Ventral side of a head with 4+4 postlabial setae. Ant.I with 15–17 common setae, two ventral s-setae (s) and three bms, one long (inseparable from common setae) and two short, Ant.II with three bms and one latero-distal s, Ant.III with one bms and with five distal s (including one lateral), without additional s-setae (Fig. 46). Several tubular s-setae on Ant.IV. Organite stick-like, small.

**Figures 40–49.**
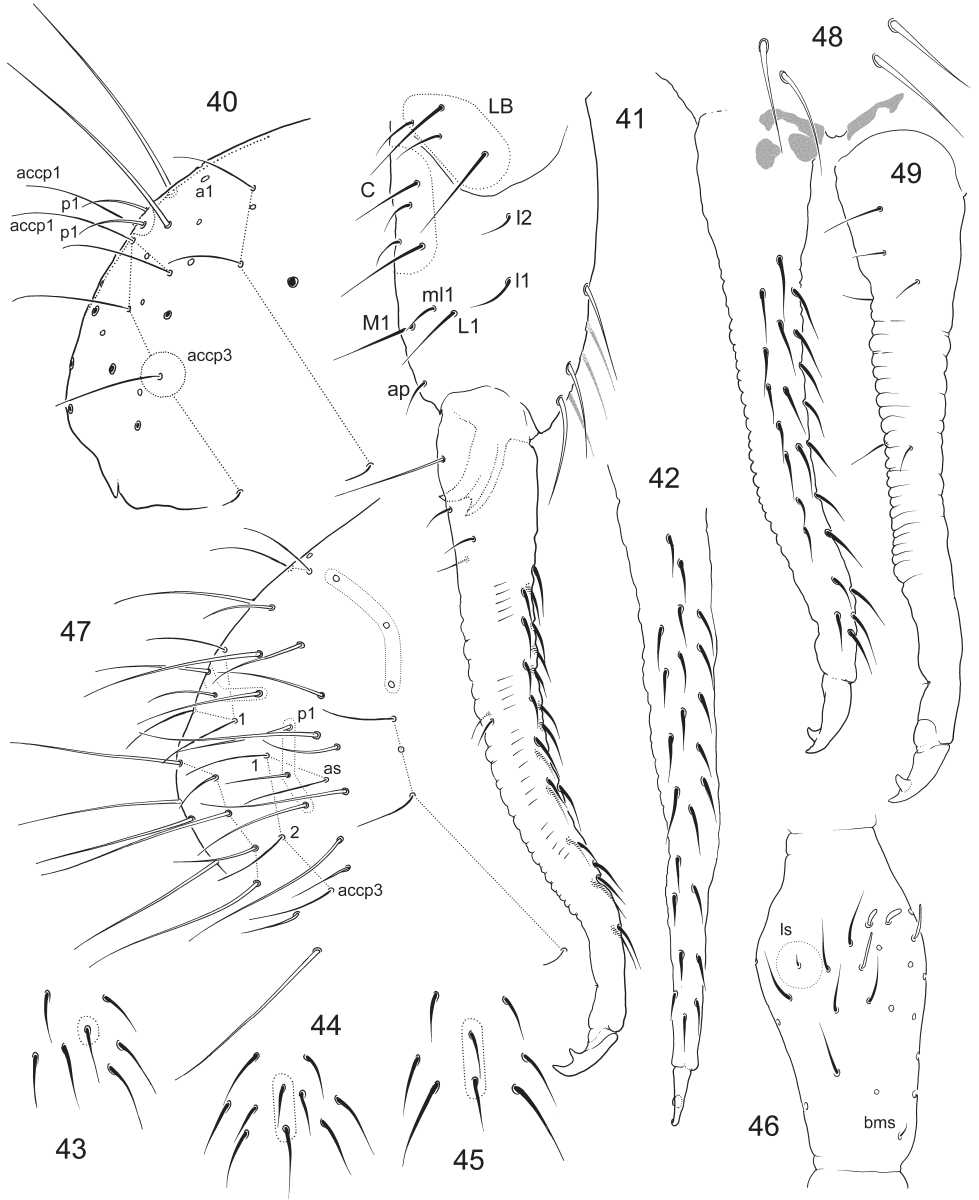
*F. imparis* sp. n. (**40–46**) and *F. macrochaetosa* (**47–49**) **46** End of abdomen, lateral view **41** Furca, lateral view (unpaired setae marked with grey) **42** Anterior side of dens **43–45** Chaetotaxy of anterior side of furca, variations **46** Ant.III **47** End of abdomen **48** Furca, anterior view **49** Dens, posterior view. Notation of setae of posterior and lateral sides of manubrium: ap, M1, ml1, L1, l1, l2; LB–latero-basal setae, C–setae of central area.

Common setae long and sparse. Sensillary formula as 43/22235 (s), 10/100 (ms). Tergal s-setae thin and long. Medial s-setae on Th.II–Abd.III situated in posterior position, on Abd.I–III between Md and Mdl. Abd.V with five s-setae arranged as four ones (as, accp1, accp2, accp3), long and slender, and one latero-ventral, short (‘4+1’ pattern) (Figs 32, 40), accp3 s-setae almost as long as accp2 (accp2 : accp3 = 1.0–1.2). p1-setae on Abd.V short (see also the Discussion and Table 2). Macrosetae smooth and long, 2,2/3,3,3 in number, medial ones on Abd.V 1.5–2.0 times shorter than dens and 4.8–7.7 times longer than mucro. Metathorax with 3+3 (rarely, 4+3) ventral setae, as in *F. amurica* sp. n.

Unguis of normal shape, without lateral and inner teeth. Empodial appendage as long as 0.4–0.5 unguis. Tibiotarsi with 26–28 setae on legs I–II and 33–37 on leg III. Upper and lower subcoxae of legs I–III with 0,1/3,~7/5–6,7–8 setae, respectively. Coxae of leg I with three front setae. Tibiotarsal tenent setae pointed, some setae on distal half of tibiotarsi thickened. Ventral tube with 4+4 latero-distal and seven posterior setae (four in distal transversal row and three in more proximal position), anteriorly without setae. Tenaculum with 4+4 teeth and a seta. Anterior furcal subcoxae with 10–12, posterior one with five setae. Anterior side of manubrium with 3–5+3–5 pair setae, and 2–3 (rarely one) axial unpaired setae (Figs 43–45). Posterior side of manubrium with 4+4 latero-basal, two apical setae (ap), 3+3 setae in distal transversal row (M1, ml1, L1), two pairs of lateral setae, and 3–4+3–4 in central part (Fig. 41). Dens with 23–27 anterior setae (Figs 41, 42). Posterior side of dens crenulated and with six normal setae (four basal and two at the middle) and one rudimentary seta at the base of mucro. Mucro bidentate. Ratio of manubrium : dens : mucro = 5.2–6.6 : 9.4–11.7 : 1. Males present.

###### Remarks.

*Folsomia imparis* sp. n. is well defined by the presence of unpaired setae on anterior side of manubrium shared only with *F. hidakana* (belongs to another subgroup) and *F. setifrontalis* (has anterior setae on ventral tube). Main differences from the other species of the subgroup ‘*macrochaetosa*’ are shown in Table 2.

A typical form, called preliminary as f. 1 (Fig. 32) has short p1-setae on Abd.I. In Japan and Primorsky Krai we often recorded specimens with long p1, as long as a1 (f. 2 in Table 2) which was not mixed with f. 1 by samples even if reported in the same areas.

Additionally, a closely related form was also recorded in two more eastern localities (see the Material part, F. sp. aff. imparis). It differs from all species listed in Table 2 having much more setae on body tergites: particularly, posterior row of Abd.IV has seven (vs. 3–4) p-setae between medial s-setae. The final decision on the status of these polychaetotic specimens was not made by us and calls for more information on their ecology and distribution.

###### Distribution and ecology.

The species is widely distributed in Far East of Russia, Korea and in the most northern part of Japan (Fig. 90). We suppose some records of *F. fimetaria* in Hokkaido refer to this species. *F. imparis* sp. n. is rather common in different forest litter, often in rotten wood.

###### Derivatio nominis.

The species has unpaired setae on anterior side of manubrium.

##### Folsomia macrochaetosa

Taxon classificationAnimaliaCollembolaIsotomidae

Martynova in Martynova, Berman & Chelnokov, 1977

Figs 47–49 , 90

###### Type Material.

Three paratypes labelled as “Magadanskaya Region, vicinities of Magadan, Snow Valley, dwarf-wood belt. 18.ix.1974. coll. Berman.” Kept in Zoological Institute (S.-Petersburg).

###### Other material.

Far East of Russia, Khabarovsky Krai, Vicinities of Nikolaevsk-na-Amure, lower flow of Amgun’ River, larch and birch forests, 1.vii.1990, coll. N. Ryabinin; Primorsky Krai, Ussuriysky Reserve, Komarovskoye Forest District, mixed forest, 22.vii.2016, coll. M. Potapov and N. Kuznetsova; Terneysky District, Sikhote-Alinski Reserve, Kabany station, hardwood with *Rhododendron*, litter, 08.viii.2017, coll. N. Kuznetsova, A. Geras’kina, A. Kuprin; South Kuril Islands, Kunashir Isl., Krugly Cape and 5 km E Yu-Kuril’sk, viii–ix.1997, coll. Y. Marusik.

South Korea, Gangwon-do, Palsan Mt., mixed forest in foothills, litter, 09.ix.2017, coll. A. Kuprin.

###### Description.

Body size from 0.9 to 1.5 mm. Body without pigmentation, cuticle with fine primary granulation. Ocelli absent. PAO slender, constricted, 1.4–1.6 as long as width of Ant.I and 1.4–2.2 as long as inner unguis length. Labium with five usual papillae (*A–E*), guard setae e7 absent, three proximal and four basomedian setae. Ventral side of a head with 4+4 postlabial setae. Ant.I with 15–16 common setae, s-setae of antennae as in *F. imparis* sp. n. Organite stick-like, small.

Common setae long. Sensillary formula as 43/22235 (s), 10/100 (ms). Tergal s-setae long, medial ones on Th.II–Abd.III situated in posterior position. Abd.V with five s-setae arranged as four ones (as, accp1, accp2, accp3), long and slender, and one latero-ventral, short (‘4+1’ pattern), accp3 s-setae somewhat shorter than accp2 (accp2 : accp3 = 1.1–1.5). p1-setae on Abd.V long (Fig. 47, see also the Discussion and Table 2). Macrosetae smooth and long, 2,2/3,3,3 in number, medial ones on Abd.V 1.3–1.7 times shorter than dens and 4.6–6.4 times longer than mucro. Metathorax with 3+3 ventral setae, one long.

Unguis of normal shape. Empodial appendage as long as 0.50–0.55 of unguis. Chaetotaxy of tibiotarsi and subcoxae similar to *F. amurica* sp. n. and *F. imparis* sp. n. Number of front setae on coxae of leg I varies (2–3), variant with two setae common for specimens from Magadan and Kunashir. Ventral tube with 4+4 latero-distal and 6–8 posterior setae, anteriorly without setae. Tenaculum with 4+4 teeth and a seta. Anterior furcal subcoxae with 11–12, posterior one with five setae. Anterior side of manubrium with 2–3+2–3 pairs of setae (2+2 usually in males), without axial unpaired setae (Figs 48–49). Dens with 21–30 anterior setae. Posterior side of dens crenulated and with six normal setae and one rudimentary at the base of mucro. Mucro bidentate. Ratio of manubrium : dens : mucro = 4.3–5.7 : 6.4–10.0 : 1. Males present.

###### Remarks.

*F. amurica* sp. n., *F. imparis* sp. n., *F. macrochaetosa*, and *F. setifrontalis* combine a group of species with long macrosetae and furca, sparse setae covering, and posterior position of median s-setae on all tergites. The differences between the species of this subgroup are shown in Table 2.

**Table 2.** Differentiated characters of three species of the ‘*macrochaetosa*’ subgroup.

Species	Abd.V : accp3-s	Abd.V : a1:p1	Paired setae on manubrium	Unpaired setae on manubrium
*F. amurica* sp. n.	short	0.9–1.1	4–6+4–6	absent
*F. imparis* sp. n.	long	f.1: 1.4–2.3 f.2: 1.0–1.2	3–5+3–5	2–3 (1)
*F. macrochaetosa*	long	1.1–1.3	2(3)+2(3)	absent
*F. setifrontalis*	long	1.6–2.3	3–5+3–5	0–2

###### Distribution.

Scattered records all over the coastal areas from Magadan (Russian Far East) to South Korea (Fig. 90).

##### Folsomia setifrontalis

Taxon classificationAnimaliaCollembolaIsotomidae

Potapov & Marusik, 1977

###### Material.

Far East of Russia, Primorsky Krai, Partizansky District, vicinities of Ekaterinovka, Chondalaz (=Lazovy) Range, oak litter, 26.ix.2004, coll. M. Potapov, L. Deharveng, R. Pomorski, and A. Bedos; Shkotovsky District, vicinities of Anisimovka and near trail to Khualaza Mount, forest litter, 10-12.ix.2001, coll. M. Potapov, Y. Bu and H. Cheng-Wang; Terneysky District, Sikhote-Alimski Reserve, Blagodatny station, oak wood on slope, rotten wood, 07.viii.2017, coll. N. Kuznetsova, A. Geras’kina, A. Kuprin.

###### Remarks.

*Folsomia setifrontalis* is sharply defined by the presence of anterior setae on ventral tube that is a unique character for the genus. Populations from Primorsky Krai differ from the type specimens (South Kuril Islands) by the presence of unpaired setae on manubrium and longer accp3-s on Abd.V. Considering this variability, a wider diagnosis is proposed for the species. So far, the chaetotaxy of ventral tube remain a key characteristic of this species.

###### Distribution and ecology.

Less common than the sympatric *F. imparis* sp. n. and *F. macrochaetosa*. Rare records in forest litter of southern part of Far East of Russia.

#### Subgroup ‘*laconica*’

##### Folsomia laconica

Taxon classificationAnimaliaCollembolaIsotomidae

Potapov & Kuznetsova sp. n.

http://zoobank.org/D39223CD-C85B-478F-9392-AB44DF1C64A9

Figs 6 , 51 , 52 , 54 , 55–57 , 90

###### Type material.

Holotype, female, Far East of Russia, Amurskaya Region, Khingansky Reserve, near (~6 km W) Kundur, valley of Karapcha River, northern steep slope, mixed forest with *Abies*, litter, 19.viii.2014, coll. M. Potapov and N. Kuznetsova. 15 paratypes from the same location and ten paratypes from Khingansky Reserve, ~10 km E Uril, coniferous forest (*Pinus koraiensis*, *Abies*, *Picea*), 7.x.2009, coll. M. Babykina. Deposited in MSPU.

###### Other material.

Amurskaya Region, Khingansky Reserve, ~15–20 km SE Uril, oak wood litter, 8.x.2009, coll. M. Babykina. North Korea, Yanggang-do Province, ~1.5 km SE Mupho, litter under *Rhododendron* and *Alnus*, 5.vii.1985, coll. A. Szeptycki.

###### Diagnosis.

Blind. Dorsal macrosetae (Md) present on both Th.II and Th.III. Sensillary formula incomplete (33/22224; 10/000). Medial s-setae on body tergites long, set in p-row. Ventral setae on Th.III present. Anterior side of manubrium with 3+3 setae, no unpaired axial setae present. Dens with 17–20 anterior setae, its posterior side with three setae in basal part. Mucro bidentate.

###### Description.

Body size approximately 1.3 mm (Fig. 51). Without pigmentation. Cuticle with fine primary granulation (“smooth”). Ocelli absent. PAO slender, not constricted or slightly constricted, 1.5–1.7 as long as width of Ant.I and 1.7–2.0 as long as inner unguis length. Labium complete, guard setae e7 present, three proximal and four basomedian setae. Ventral side of head with 4+4 postlabial setae. Ant.I with 16–17 common setae, two ventral s-setae (s) and three basal micro s-setae (bms): two dorsal (short and long) and one ventral. Ant.II with three bms and one latero-distal s, Ant.III with one bms and with five distal s (including one lateral), without additional s-setae. Ant.IV with stick-like organite.

Common setae sparse, macrosetae long (Fig. 51). Sensillary formula as 33/22224 (s), three s-setae lost: corner accp-s on Th.II, one of dorsal accp-s on Abd.IV, and as-s on Abd.V (Figs 55–56). Micro s-setae as 10/000 (ms). Tergal s-setae thin and long, lateral s-setae on abdomen shorter. Medial s-setae on Th.II–Abd.III situated in posterior position, on Abd.I–III between Md and Mdl. Abd.V with four s-setae arranged as three long and slender (accp1, accp2, accp3) and one latero-ventral, short (‘3+1’ pattern) (Figs 52, 57), accp3 s-setae as long as accp2 (ratio accp2 : accp3 = 1.0–1.2). Macrosetae smooth and very long, 2,2/3,3,3 in number, medial ones on Abd.V slightly shorter than dens (dens: Md = 1.0–1.3) and 7.3–9.3 times longer than mucro. Metathorax with 3+3 ventral setae.

Unguis of normal shape, without lateral and inner teeth. Empodial appendage as long as ~0.5 of unguis. All tibiotarsi with additional setae: 28–29 on legs I–II and >35 on leg III. Upper and lower subcoxae of legs I–III with 0,1/3,7/6,8 setae, respectively. Coxae of leg I with three front setae. Tibiotarsal tenent setae pointed, few setae on distal half of tibiotarsi III slightly thickened. Ventral tube with 4+4 latero-distal and 6–7 posterior setae (4 in distal transversal row), anteriorly without setae. Tenaculum with 4+4 teeth and a seta. Anterior furcal subcoxae with 12–14, posterior one with five setae. Anterior side of manubrium with 3+3 setae (Fig. 54). Posterior side of manubrium with 5+5 latero-basal, two apical setae (ap), 2+2 setae in distal transversal row (M1, ml1), two pairs of lateral setae, and 4+4 in central part (Fig. 54). Dens with 17–20 anterior setae. Posterior side of dens crenulated and with six setae: three basal, two at the middle, and one subapical (Fig. 54). Subapical seta often hardly visible. Mucro bidentate. Ratio of manubrium : dens : mucro = 5.4–7.2 : 7.8–9.3 : 1.

###### Remarks.

*Folsomia laconica* sp. n. most resembles *F. trisensilla* sp. n. but differs having three (vs four) basal setae on posterior side of dens) (Figs 54, 63–64) and fewer common setae at the end of abdomen. The latter character is expressed in 2+2 dorsal p-setae on Abd.V (vs 3+3 in *F. trisensilla* sp. n.) and 2–3 p-setae between s-setae on Abd.IV (vs 4–5) (Figs 52, 53).

**Figures 50–54.**
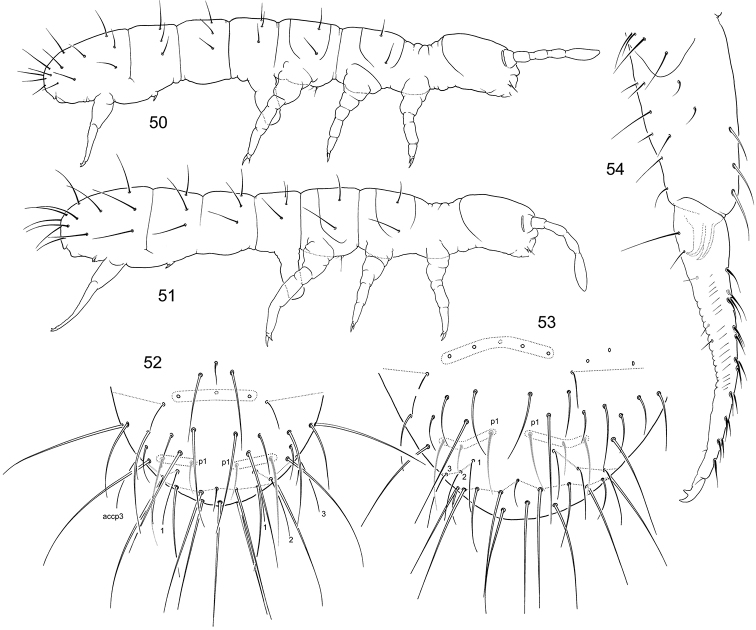
*F. trisensilla* sp. n. (**50, 53**) and *F. laconica* sp. n. (**51, 52, 54**) **50–51** Appearance **52–53** End of abdomen, dorsal view **54** Furca, lateral view.

**Figures 55–57.**
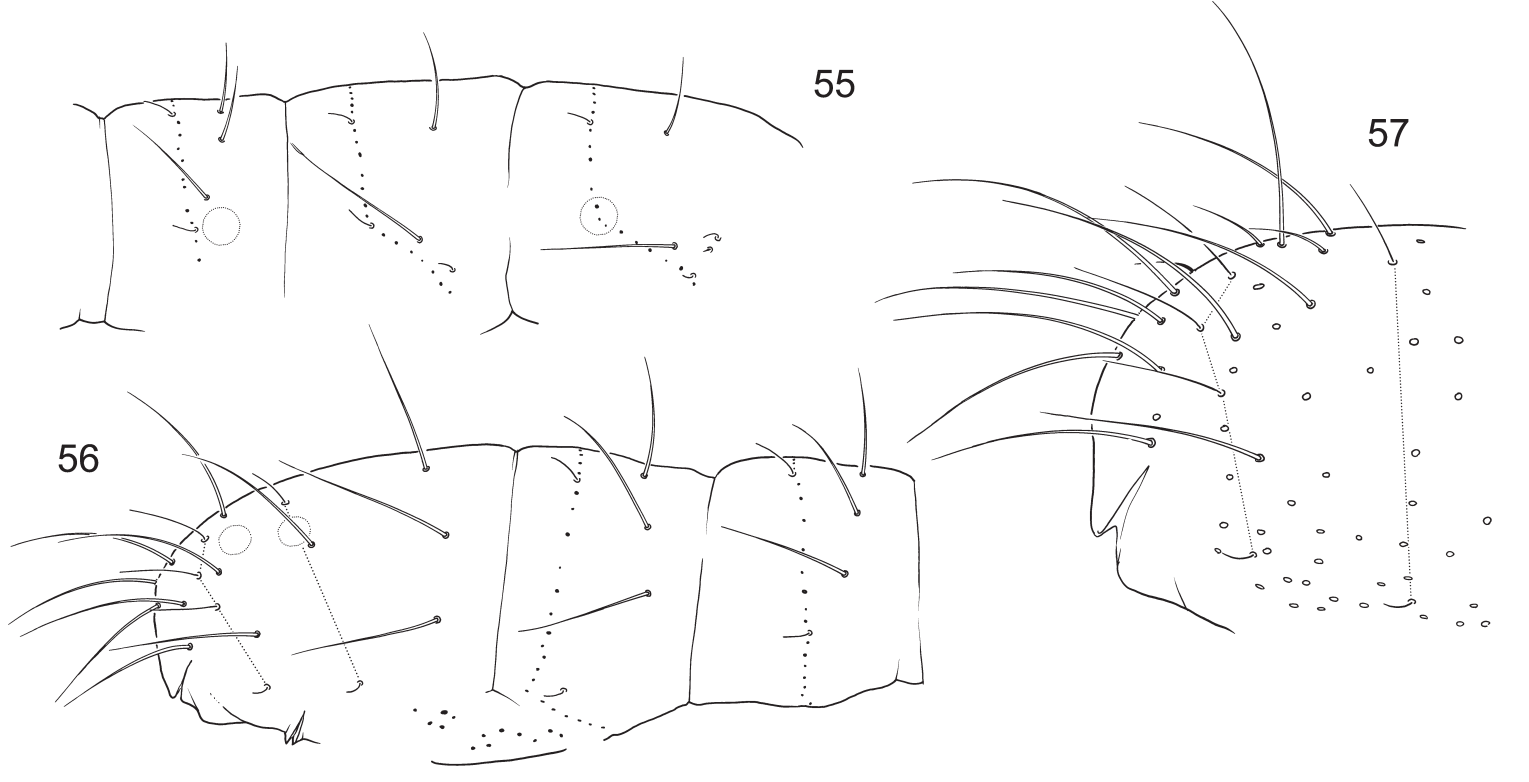
*F. laconica* sp. n. **55–56** Position of macrosetae, setae of p-row, and s-setae on anterior (**55**) and posterior (**56**) half of corpus **57** End of abdomen, lateral view.

###### Distribution and ecology.

Known from three locations in Russian Far East and North Korea (Fig. 90). It occurs in forest litter.

###### Derivatio nominis.

The species shows the most laconic chaetotaxy of p-row on Abd.V.

##### Folsomia trisensilla

Taxon classificationAnimaliaCollembolaIsotomidae

Potapov sp. n.

http://zoobank.org/004AE2D2-2ED2-4C31-AB69-BD8792B7FBAD

Figs 50 , 53 , 58–64 , 90

###### Type material.

Holotype, female, Far East of Russia, Khabarovsky Krai, Vaninsky District, 10 km N Vysokogorny, valley of Mulinka River, closed *Picea* and *Abies* forest on NE slope, litter, ~ 750 m alt., 29.ix.2011, coll. M. Potapov. Ten paratypes from the same location and six paratypes from the nearly same location, in litter of coniferous forests at different altitudes (600 and 900 m alt.), 29.ix.2011, coll. M. Potapov. Deposited in MSPU.

###### Other material.

Far East of Russia, Khabarovsky Krai. Different sites of type locality, litter and rotten wood, 29.ix.2011, coll. M. Potapov; Kamchatka. Nearby Anavgai and Esso settlements, different larch forests, litter and rotten wood, ~ 500 m alt., 4.vii.2012, coll. M. Potapov; Primorsky Krai. Terneysky District, Sikhote-Alimski Reserve, Kabany station, forest litter, 08.viii.2017, coll. N. Kuznetsova, A. Geras’kina, A. Kuprin; East Siberia: Chitinskaya Region, near foothills of Daursky Range, valley of Ilya River, ~ three km station Ara-Ilya, 50.9253°N, 113.1783°E, 887 m alt., mixed forest with *Betula* and *Larix*, 11.vii.2014, coll. A. Gulgenova.

###### Diagnosis.

Blind. Dorsal macrosetae (Md) present on both Th.II and Th.III. Sensillary formula incomplete (33/22224; 10/000). Medial s-setae on body tergites long, set in p-row. Ventral setae on Th.III present. Anterior side of manubrium with 3+3 paired setae, no unpaired axial setae present. Dens with 12–16 anterior setae, its posterior side with four setae in basal part. Mucro bidentate.

###### Description.

Body size from 1.0 to 1.3 mm, rather tubular (Fig. 50). Without pigmentation. Cuticle “smooth”. PAO slender, slightly constricted, 1.5–1.7 as long as width of Ant.I and 1.7–2.0 as long as inner unguis length (Fig. 62). Labium complete, guard setae e7 present, three proximal and four basomedian setae. Ventral side of head with 4+4 postlabial setae. Ant.I with 15–16 common setae, two ventral s-setae (s) and three basal micro s-setae (bms): two dorsal (short and long) and one ventral. Ant.II with three bms and one latero-distal s, Ant.III with one bms and with five distal s (including one lateral), without additional s-setae. Several s-setae on Ant.IV tubular. Organite middle-sized, roundish.

**Figures 58–64.**
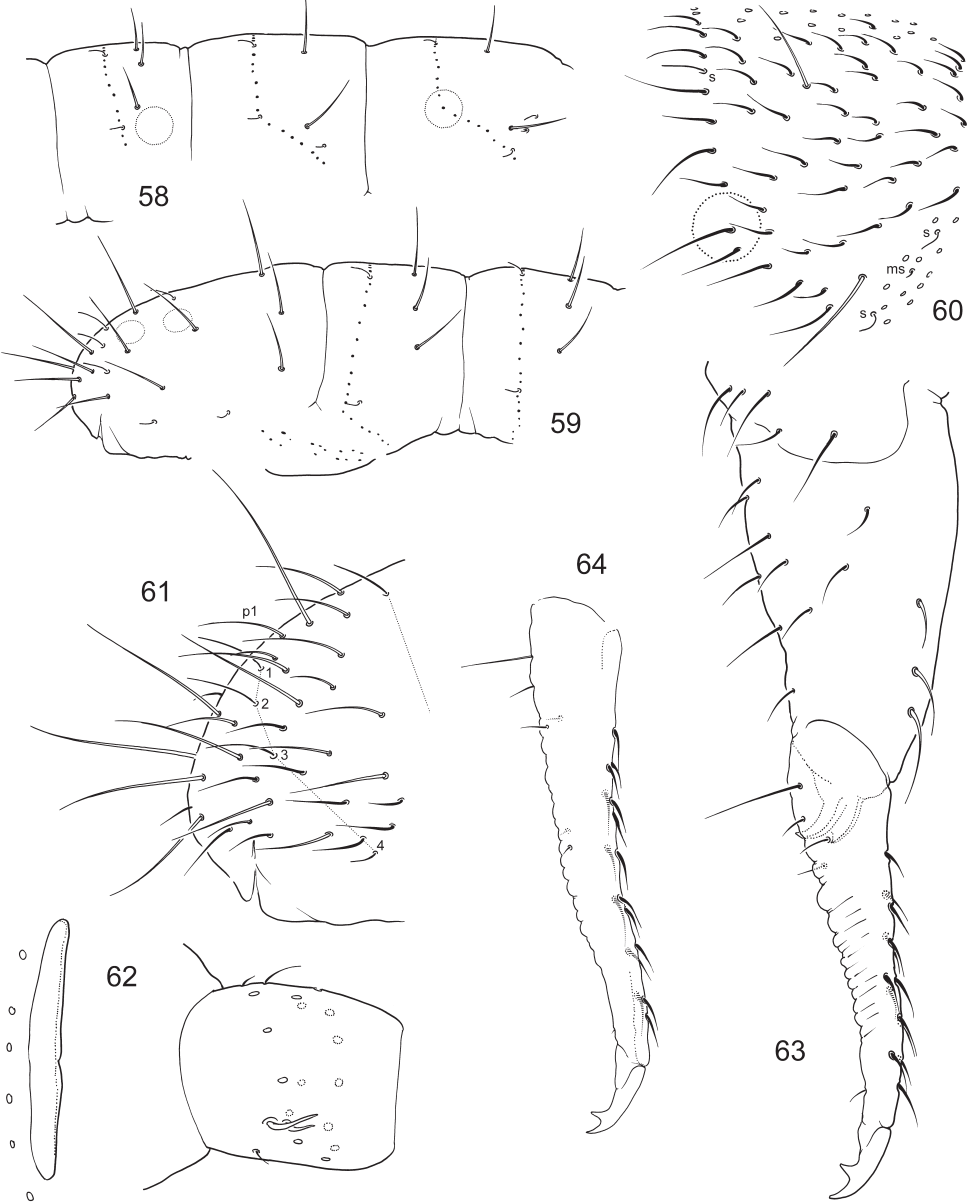
*F. trisensilla* sp. n. **58–59** Position of macrosetae, setae of p-row, and s-setae on anterior (**58**) and posterior (**59**) half of corpus **60** Th.II **61** End of abdomen **62** PAO and Ant.I **63** Furca, lateral view (type population) **64** Dens, lateral view (Kamchatka).

Common setae long. Sensillary formula as 33/22224 (s), three s-setae lost (as described for the ‘*laconica*’ subgroup (Figs 58, 59). Corner s-setae on Th.II absent (Fig. 60). Micro s-setae as 10/000 (ms). Tergal s-setae thin and long, lateral s-setae on abdomen shorter. Medial s-setae on Th.II–Abd.III situated in posterior position, on Abd.I–III between Md and Mdl. Abd.V with four s-setae arranged as three long and slender (accp1, accp2, accp3) and one latero-ventral, short (‘3+1’ pattern) (Figs 53, 61), accp3 s-setae as long as accp2 (0.9–1.1). Macrosetae smooth and long, 2,2/3,3,3 in number, medial ones on Abd.V slightly shorter than dens (1.0–1.3) and 3.5–4.6 times longer than mucro. Metathorax with 3+3 ventral setae.

Empodial appendage as long as 0.5–0.6 of unguis. All tibiotarsi with additional setae: 24–26 on legs I–II and 30–35 on leg III. Upper and lower subcoxae of legs I–III with 0,1/3,7–8/6–7,7–8 setae, respectively. Coxae of leg I with three (rarely two on one side) front setae. Tibiotarsal tenent setae pointed, some setae of distal whorl of tibiotarsi thickened. Ventral tube with 4+4 latero-distal and 7–8 posterior setae (four in distal transversal row and 3–4 in more proximal position), anteriorly without setae. Tenaculum with 4+4 teeth and a seta. Anterior furcal subcoxae with 8–10, posterior one with four setae. Anterior side of manubrium with 3+3 setae (rarely 2+3) (Fig. 63). Posterior side of manubrium with 5(4)+5(4) latero-basal, two apical setae (ap), 2+2 setae in distal transversal row (M1, ml1), two pairs of lateral setae, and 4–6+4–6 in central part (Fig. 63). Dens with 12–16 anterior setae. Posterior side of dens crenulated and with five setae (4 basal and one shorter one at the base of mucro), two setae at the middle present or absent (Figs 63, 64, see also the Discussion part). Mucro bidentate. Ratio of manubrium : dens : mucro = 4.1–5.0 : 3.9–5.2 : 1.

###### Remarks.

Two setae at the middle of posterior side of dens are always absent in the type populations (Fig. 63, Khabarovsky Krai). Specimens from Kamchatka possess these setae (always rudimentary and one sometimes asymmetrically lost) (Fig. 67). We accept a wide diagnosis of the new species considering both variants.

An odd population was found near Uril (Amurskaya Region) differing from the typical ones by having 4+4 (vs 3+3) anterior setae on manubrium (left out of diagnosis of the new species so far).

For difference between *F. trisensilla* sp. n. and *F. laconica* sp. n. see Remarks to the latter.

###### Distribution and ecology.

Known from forest litter in three localities in Eastern Asia (Fig. 90).

###### Derivatio nominis.

The new species name reflects the presence of three (vs four, as common for the genus) s-setae on dorsal side of Abd.V that is characteristic of the ‘*laconica*’ subgroup.

##### Folsomia tertia

Taxon classificationAnimaliaCollembolaIsotomidae

Potapov sp. n.

http://zoobank.org/6294599B-3CEA-4971-8B72-6B28918EA511

Figs 65–68

###### Type material.

Holotype, female, Far East of Russia, Khabarovsky Krai, vicinities of Khabarovsk, Bol’shoy Khekhtsyr Range, ~10 km N Korfovsky, mixed forest litter, 28.vi.2007, coll. E. Sokolova. Ten paratypes from the same location. Deposited in MSPU.

###### Diagnosis.

Blind. Dorsal macrosetae (Md) present on both Th.II and Th.III. Sensillary formula incomplete (33/22224; 10/000). Medial s-setae on body tergites long, set in p-row. Ventral setae on Th.III present. Anterior side of manubrium with 2+2 setae, no unpaired axial setae present. Dens with 13–16 anterior setae, its posterior side with four setae in basal part. Mucro bidentate.

###### Description.

Body size from 0.9 to 1.2 mm, shape of corpus rather tubular, slender. Without pigmentation. Cuticle with fine hexagonal primary granulation (“smooth”). PAO slender, insignificantly constricted, 1.6–1.8 as long as width of Ant.I and 2.1–2.2 as long as inner unguis length. Labium complete, guard setae e7 present, three proximal and four basomedian setae. Ventral side of head with 4+4 postlabial setae. Ant.I with 14–15 common setae, two ventral s-setae (with one thick) and three basal micro s-setae (bms): two dorsal (short and long) and one ventral. Ant.II with three bms and one latero-distal s, Ant.III with one bms and with five distal s (including one lateral), without additional s-setae. Organite normal, small.

**Figures 65–68.**
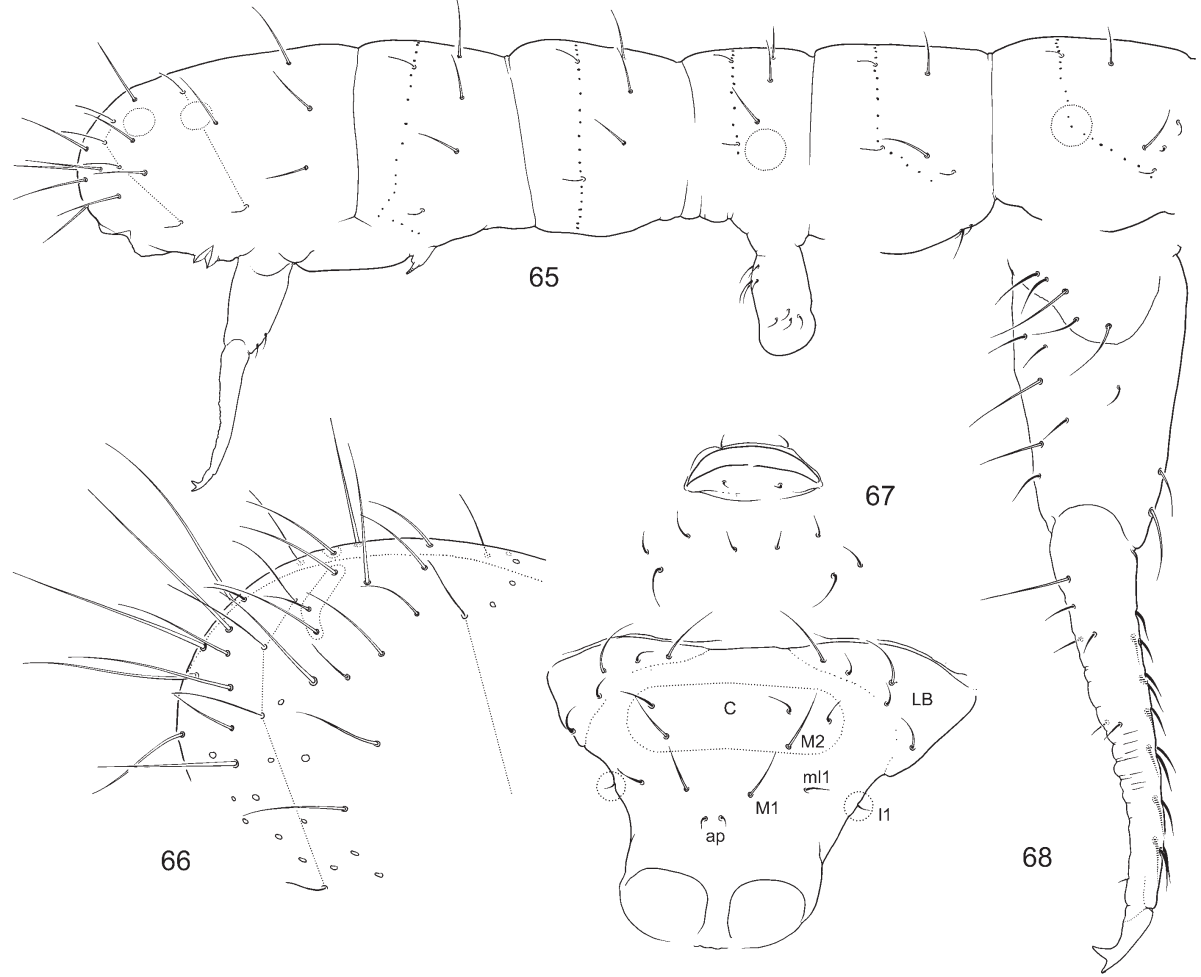
*F. tertia* sp. n. **65** Position of macrosetae, setae of p-row, and s-setae on corpus **66** End of abdomen **67** Posterior side of manubrium and genital area in adult female **68** Furca, lateral view. Notation of setae of posterior and lateral sides of manubrium: ap, M1, ml1, M2, l1; LB–latero-basal setae, C–setae of central area.

Sensillary formula as 33/22224 (s), three s-setae lost (as in other species of the subgroup, Fig. 65). Micro s-setae as 10/000 (ms). Tergal s-setae thin and long, lateral s-setae on abdomen shorter. Medial s-setae on Th.II–Abd.III situated in posterior position, on Abd.I–III between Md and Mdl. Abd.V with four s-setae arranged as three ones (accp1, accp2, accp3), long and slender, and one latero-ventral, shorter (‘3+1’ pattern) (Fig. 66), accp3 s-setae almost as long as accp2 (accp3 : accp2 = 0.8–1.0). With 3+3 medial p-setae on Abd.V: p1 and p3 long, p2 short. Seta p1 somewhat longer than a1 and accp1-s on Abd.V (a1 : p1 = 0.7–0.8, accp1 : p1 = 0.8–0.9). Macrosetae smooth and rather long, 2,2/3,3,3 in number, medial ones at the end of abdomen slightly shorter than dens (0.8–0.9) and 2.8–3.9 times longer than mucro. Metathorax with 3+3 ventral setae.

Empodial appendage as long as 0.55–0.60 of unguis. Tibiotarsi with few additional setae: 22–24 on legs I–II and ~29 on leg III. Upper and lower subcoxae of legs I–III with 0,1/3,7/3–5,~8 setae, respectively. Coxae of leg I with two front setae. Tibiotarsal tenent setae pointed, few setae of whorl of tibiotarsi insignificantly thickened. Ventral tube with 4+4 latero-distal and 5–7 posterior setae (four in distal transversal row and 1–3 in more proximal position), anteriorly without setae. Tenaculum with 4+4 teeth and a seta. Anterior furcal subcoxae with 7–9, posterior one with 5–6 setae. Anterior side of manubrium with 2+2 setae (Fig. 68). Posterior side of manubrium with 5+5 latero-basal, two apical setae (ap), 2+2 setae in distal transversal row (M1, ml1), one pair of lateral setae, and 3(2) +3(2) in central part (Fig. 67). Dens with 13–16 anterior setae. Posterior side of dens crenulated and with seven setae (four basal, two at the middle, and one very short at the base of mucro) (Figs 68). Two setae at the middle of dens often very short, one sometimes absent. Subapical seta often hardly visible. Mucro bidentate. Ratio of manubrium : dens : mucro = 3.1–4.1 : 3.3–4.5 : 1. Males present.

###### Remarks.

The new species belongs to the ‘*laconica*’ subgroup due to incomplete s-set on body. It differs from two other members, *F. laconica* sp. n. and *F. trisensilla* sp. n., by reduced chaetotaxy on anterior (2+2 instead of 3–4+3–4 setae) and posterior sides (1+1 vs 2+2 lateral setae) of manubrium. *F. tertia* sp. n. has the shortest macrosetae among species of the subgroup and somewhat resembles members of the ‘*tatarica*’ subgroup.

###### Distribution.

Known only from type locality.

###### Derivatio nominis.

The new species is the third (*tertius* in Latin) species of the ‘*laconica*’ subgroup.

#### Subgroup ‘*tatarica*’

##### Folsomia calcarea

Taxon classificationAnimaliaCollembolaIsotomidae

Potapov sp. n.

http://zoobank.org/E4AB1B52-2108-4802-BC00-A8843C5F3372

Figs 69 , 71–75

###### Type material.

Holotype, female, Russia, European part, Samarskaya District, Samarskaya Luka National Park, calcareous stone-pit, soil, 02.v.2010, coll. Y. Shveenkova. Six paratypes from the same location and maple forest nearby. Deposited in MSPU.

###### Diagnosis.

Blind. Body slender. Dorsal macrosetae (Md) present on both Th.II and Th.III. Sensillary formula incomplete (43/22235; 10/000). Medial s-setae on body tergites in anterior position. Th.III with 1+1 ventral setae. Anterior side of manubrium with 1+1 setae, dens with 6–7 anterior setae. Mucro bidentate.

###### Description.

**Figures 69–75.**
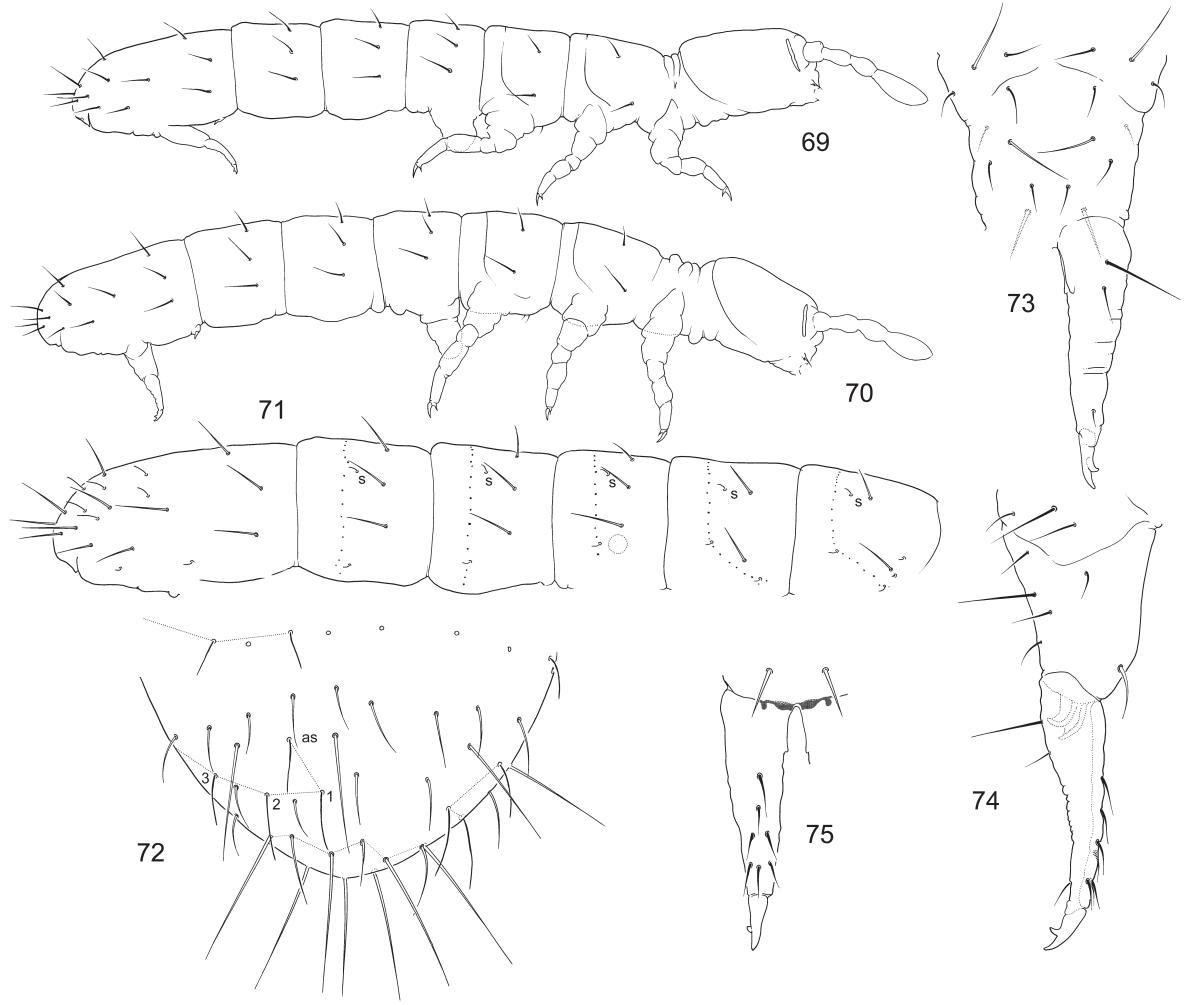
*F. calcarea* sp. n. (**69, 71–75**) and *F. tubulata* sp. n. (**70**) **69–70** Appearance **71** Position of macrosetae, setae of p-row, and s-setae on corpus **72** End of abdomen, dorsal view **73–75** Furca, posterior (**73**), lateral (**74**), and anterior (**75**) views.

Body size near 1.1 mm (for the largest subadult female). Body slender, tubular (Fig. 69). Cuticle with fine hexagonal primary granulation (“smooth”). Ocelli absent. PAO slender, constricted, 1.8–2.5 as long as width of Ant.I and 2.3–2.7 as long as inner unguis length. Labium with five usual papillae (*A–E*), guard setae e7 absent, three proximal and four basomedian setae. Ventral side of head with 3+3 postlabial setae. Ant.I with 11–12 common setae, two ventral s-setae (s) and three basal micro s-setae (bms), two dorsal (middle-sized and short) and one ventral (short), Ant.II with three bms and one latero-distal s, Ant.III with one bms and with four distal s (lateral s absent), without additional s-setae. Organite short and small.

Common setae short. Sensillary formula as 43/22235 (s), 10/000 (ms) (Fig. 71). Tergal s-setae thin and hardly differ from common seta, longer on dorsal side of Abd.V. Medial s-setae on Th.II–Abd.III situated in mid-tergal position, on Abd.I–III between Md and Mdl. Abd.V with five s-setae arranged as four ones (as, accp1, accp2, accp3), long and slender, and one latero-ventral, short (‘4+1’ pattern) (Fig. 72), accp3 s-setae almost as long as accp2 (0.9–1.2). Macrosetae smooth, 2,2/3,3,3 in number, medial ones on Abd.V as long as 0.8–1.3 of dens and 2.6–3.3 times longer than mucro. Metathorax with 1+1 ventral setae.

*Unguis* of normal shape, without lateral and inner teeth. Empodial appendage as long as 0.4–0.5 of unguis. Tibiotarsi with few setae: 21–22 on legs I–II and 24–27 on leg III. Upper and lower subcoxae of legs I–III with 0,1/3,5–7/4–5,6–6 setae, respectively. Coxae of leg I with two front setae. Ventral tube with 4+4 latero-distal and 4–5 posterior setae (four in distal transversal row), anteriorly without setae. Tenaculum with 4+4 teeth and a seta. Anterior furcal subcoxae with 3–4, posterior one with three setae. Anterior side of manubrium with 1+1 setae (Fig. 75). Posterior side of manubrium with 3+3 latero-basal, 4+4 on main part, and 1+1 setae on lateral side (Fig. 73). Dens with seven (rarely six) anterior setae (Figs 74 and 75). Posterior side of dens slightly crenulated and with three setae (two basal and one at the base of mucro. Mucro bidentate. Ratio of manubrium : dens : mucro = 2.8–3.9 : 2.6–3.4 : 1.

###### Remarks.

Two key characters, short furca and anterior position of medial s-setae on body tergites, are shared with only *F. torpeda*. *F. calcarea* sp. n. differs by ms-setae on Abd.I missing (vs present in *F. torpeda*) and fewer number of setae on posterior side of manubrium. The absence of lateral s on Ant.III is shared only with *F. breviseta* sp. n. but value of this characteristic requires further study.

###### Distribution and ecology.

Known only from the type locality. The species possibly belongs to calciphilous fauna.

###### Derivatio nominis.

The species was recorded in calcareous soil.

##### Folsomia tubulata

Taxon classificationAnimaliaCollembolaIsotomidae

Potapov & Babenko sp. n.

http://zoobank.org/854D6C06-9C07-4F55-A31D-50E5DA94D4D4

Figs 70 , 76–80

###### Type material.

Holotype, adult male, Russia, East Siberia, Yakutia (Sakha Republic), Lensky District, Chayandinsky allotment, July of 2011, coll. V. Boeskorov. Two paratypes from the same location. Deposited in MSPU.

###### Diagnosis.

Blind. Body slender. Dorsal macrosetae (Md) present on both Th.II and Th.III. Sensillary formula complete (43/22235; 10/100). Medial s-setae on body tergites set in p-row. Th.III with 2+2 ventral setae. Anterior side of manubrium with 1+1 setae, dens with 5–7 anterior setae. Mucro bidentate.

###### Description.

Body size 0.8–0.9 mm. Body slender, tubular (Fig. 70). Cuticle orthogonal, finely reticulated. Ocelli absent. PAO slender, not constricted or slightly constricted, 1.5–1.8 as long as width of Ant.I and 2.3–2.4 as long as inner unguis length (Fig. 78). Labium with five usual papillae (*A–E*), guard setae e7 present, three proximal and four basomedian setae. Ventral side of head with 3–4+3–4 postlabial setae. Ant.I with eleven common setae, two ventral s-setae (s) and three basal micro s-setae (bms) (Fig. 78), two dorsal (middle-sized and short) and one ventral (short), Ant.II with three bms and one latero-distal s, Ant.III with one bms (not found in one specimen) and with five distal s (including one lateral), without additional s-setae. Organite short and small.

**Figures 76–80.**
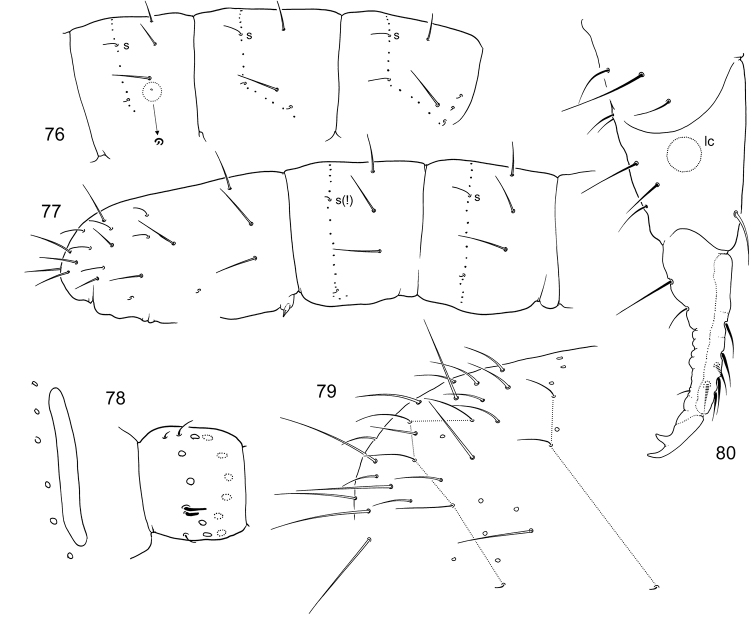
*F. tubulata* sp. n. **76** Position of macrosetae, setae of p-row, and s-setae on anterior (**76**) and posterior (**77**) half of corpus **78** PAO and Ant.I **79** End of abdomen, lateral view **80** Furca, lateral view (potential area of latero-central setae on manubrium marked).

Common setae short. Sensillary formula as 43/22235 (s). Medial s-setae on Th.II–Abd.III situated in p-row, on Abd.I–III between Md and Mdl (Figs 76, 77). Tergal s-setae vary in length depending on position. Long s-setae: two accp-s on Th.II, medial accp-s on Th.III–Abd.II. Short s-setae: all as-s on Th.II–III, corner accp-s on Th.III, lateral accp-s on Abd.I–III, medial accp-s on Abd.III. Two dorsal accp-s on Abd.IV long, latero-ventral short. Abd.V with five s-setae arranged as four (as, accp1, accp2, accp3), long and slender, and one latero-ventral short (‘4+1’ pattern) (Figs 76, 77, 79), accp3 s-setae as long as accp2. Micro s-formula as 10/100 (ms), but ms-setae of Abd.I very small and hardly visible (Fig. 76). Macrosetae smooth and short, 2,2/3,3,3 in number, medial ones on Abd.V as long as 0.9–1.0 dens and 2.4–2.9 times longer than mucro. Metathorax with 2+2 ventral setae.

Empodial appendage as long as 0.4–0.5 of unguis. Tibiotarsi with 22 setae on legs I–II and 25–27 on leg III. Upper and lower subcoxae of legs I–III with 0,1/1,6–7/3–4,5–6 setae, respectively. Coxae of leg I with two front setae. Ventral tube with 4+4 latero-distal and three (seen in only one specimen) posterior setae, anteriorly without setae. Tenaculum with 4+4 teeth and a seta. Anterior furcal subcoxae with 6–7, posterior one with three setae. Anterior side of manubrium with 1+1 setae (Fig. 80). Posterior side of manubrium with 3+3 latero-basal, 4–5+4–5 on main part, and without setae on lateral sides (Fig. 80). Dens with 5–7 anterior setae. Posterior side of dens slightly crenulated and with three setae (two basal and one at the base of mucro. Mucro bidentate. Ratio of manubrium : dens : mucro = 3.0–3.5 : 2.3–2.5 : 1. Males present.

###### Remarks.

Within ‘*tatarica*’ group, only *F. baida* (Ural Mts.) has medial s-setae in posterior position on all tergites. *F. tubulata* sp. n. shows more reduced furca: one pair of anterior setae on manubrium (vs two pairs in *F. baida*), latero-central setae on manubrium absent (vs present), 5–7 setae on anterior side of dens (vs 9–10). Similar structure of furca is known for *F. tatarica* (steppe zone of Eastern Europe) which has another s-pattern on tergites (Table 1). Strong reduction of ms-setae on Abd.I is uncommon while calls for further study.

###### Distribution.

Known from the type locality.

###### Derivatio nominis.

The species is named after tubular shape of body.

##### Folsomia inoculata

Taxon classificationAnimaliaCollembolaIsotomidae

Stach, 1947

Figs 7 , 81–89 , 90

Syn.: Folsomia ezoensis Yosii, 1965

###### Type material of J. Stach.

Two adult females from the collection of J. Stach labelled as “Polonia, Czarhohora, 28.VI.1922, leg. Smraczynski. *F. inoculata*”. Kept in the Institute of Systematics and Evolution of Animals, Polish Academy of Sciences in Krakow, Poland.

###### Additional material.

East Asia. Japan, Honshu Island. Nagano Prefecture, E Chino city, Kitayama, surroundings of Mugikusa Hutte, 2255 m alt., 36.0404°N, 138.3679°E, coniferous green moss forest with *Tsuga*, north slope, litter, 10.viii.2016; ibidem, 36.0653°N, 138.3410°E, stony meadow, grass turf, 11.viii.2016, coll. M. Potapov and N. Kuznetsova; Hokkaido Island. Japan, Hokkaido Island, Shiretoko Peninsula, trail to Mont. Rausu, different forests, litter and rotten wood, 19.viii.2016, from 350 to 1100 m alt.; Shiretoko Peninsula, nearby Shiretoko Pass, 593 m alt., 44.0617°N, 145.0913°E, stony mixed forest with *Betula ermanii*, 17.viii.2016; Shiretoko Peninsula, surroundings of Utoro, 500-year mixed forest, rotten wood, 20.viii.2016, 97 m alt., 44.1006°N, 145.0584°E, coll. M. Potapov and N. Kuznetsova.

China: Jilin Province, 5.viii.2009, Nearby Tian Lake, Changbai Mts., 1718 m alt., coniferous forest, soil under tree, coll. D. Wu.

Far East of Russia, Primorsky Krai, Shkotovsky District, Pidan Mount, ~800 m alt., rotten wood, 20.ix.2004, coll. M.Potapov, L.Deharveng, R.Pomorski, and A. Bedos; Shkotovsky District, trail to Mont. Khualaza, deciduous forest, rotten wood. 21.vii.2016, coll. M. Potapov and N. Kuznetsova; Sakhalin, Kholmsky District, South Kamysh Ridge of the Western Sakhalin Mountains, Spamberg Mt., mixed forest on slope, litter, 15.vi.2017, coll. A. Kuprin; Yuzhno-Sakhalinsk, Susunaysky Range, Chekhov peak, litter on top, 16.vi.2017, coll. A. Kuprin; Khabarovsky Krai, Sikhote-Alin Range, Nanaisky District, ~ 15 km N road Khabarovsk-Sovetskaya Gavan, Golaya mount. massif, Studeny Pass, coniferous forest, rotten wood, 28.vi–07.vii.2017, coll. A. Brinev; Sikhote-Alin Range, Vaninsky District, nearby Vysokogorny, valley of Mulinka River, rotten wood, ~ 600 m alt., 29.ix.2011, coll. M. Potapov; Vaninsky District, nearby Datta, coastal larch-wood, 28.ix.2011, coll. M. Potapov; Kamchatka, Elizovsky District, vicinities of Malki, 53.3219°N, 157.5502°E, 260 m alt., *Betula ermanii* forest, litter and rotten wood, 26.vi.2012, coll. M. Potapov and N. Kuznetsova.

Additionally, specimens from 32 localities, i.e. Ukraine (Skolevskiye Beskids), Bosnia (Perucica), Germany (Helgoland Isl., Zittau Mts, and Bavarian Alps), France (Mont Blanc), Russia (Komi, Middle Ural Mts.), Caucasus (Teberda, Guzeripl, Tsey, Khosta, Krasnaya Polyana, Lagonaki, and several other locations in Western part of North Caucasus), Armenia (Dilizhan), Georgia (Batumi, Kutaisi), Turkey (one unprecise locality, coll. L. Deharveng), Kazakstan (West Altai), Russia, West Siberia (Altai Mts.) and East Siberia (Podkamennaya Tunguska, Shira, W Sayan Mts), were examined.

###### Description.

**Figures 81–89.**
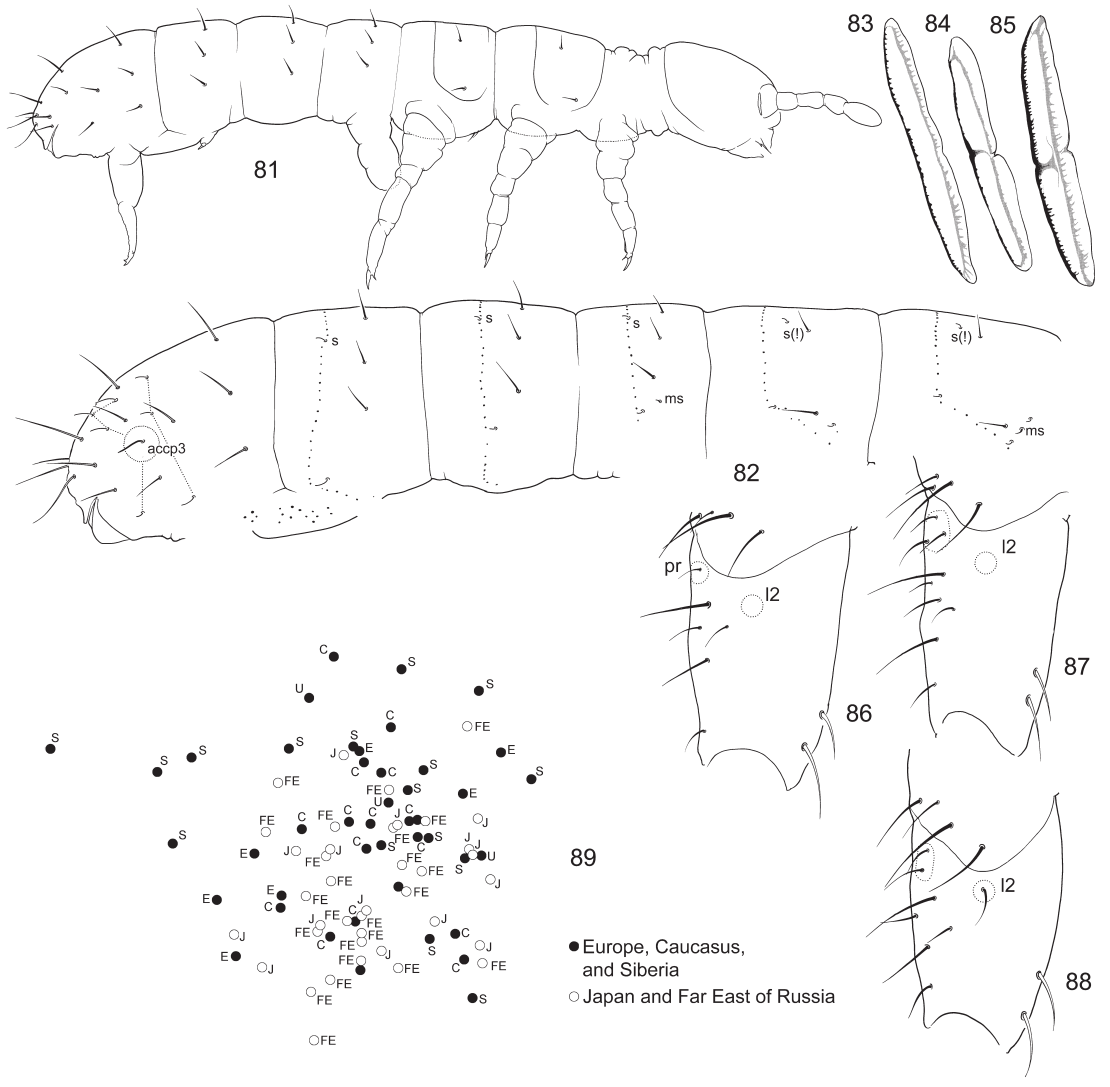
*F. inoculata* **81** Appearance **82** Position of macrosetae, setae of p-row, and s-setae on corpus **83** PAO, variations (**83, 84** Japan **85** Caucasus) **86–88** Manubrium, lateral view (Germany: Helgoland, coll. J. Schulz (**86**), Caucasus: Aibga (**87**), Japan: central Honshu (**88**) **89** Scatterplot of 84 individuals from five large regions of Palearctic basing on nine length ratios (for explanations see the Methods part). Abbreviations: pr, l2–groups of setae on posterior side of manubrium (notation as in Fjellberg, 2007), C–Caucasus, E–Europe, FE–Far East of Russia, J–Japan, S–Siberia, U–Ural.

Body stout, very characteristic, head massive, with swollen front (Fig. 81) and brown robust mouth parts. Size from 0.9 to 1.7 mm. Without pigmentation. Cuticle with fine hexagonal primary granulation. PAO slender, usually constricted, often with small “inner denticles” (Figs 83–85) (see also the Discussion part). PAO length 1.1–1.7 as long as width of Ant.I and 1.3–2.1 as long as inner unguis length. Labium complete, guard setae e7 present, three proximal and four basomedian setae. Mandible and maxillary head strongly sclerotized. Ventral side of head with 4+4 postlabial setae. Ant.I with 13–15 common setae, two ventral s-setae (s) and three basal short micro s-setae (bms). Ant.II with three bms and one latero-distal s, Ant.III with one bms and with five distal s (including one lateral), without additional s-setae.

Common setae short. Sensillary formula as 43/22235 (s). Micro s-setae as 10/100 (ms). Tergal s-setae short and distinct. Medial s-setae on Th.II–III in front of p-row, on Abd.I–III in posterior position, between Md and Mdl. Abd.V with five s-setae arranged as three short (as, accp1, accp2), one lateral long and tubular, and one latero-ventral, short (‘3+1+1’ pattern) (Fig. 82), accp3 s-setae much longer than accp2 (accp2:accp3=0.5–0.9). Macrosetae smooth and short, 2,2/3,3,3, medial ones on Abd.V shorter than dens, with the whole range of ratio Mac : dens as 0.6–1.1, and 1.9–3.1 times longer than mucro. Foil setae at the tip of abdomen absent. Thorax with 2–4+2–4 subequal setae at ventral line.

Unguis of normal shape, without lateral and inner teeth. Empodial appendage usually longer than half of unguis (0.5–0.7). All tibiotarsi with additional setae: 23–27 setae on legs I–II and >30 setae on leg III, as a whole. Upper and lower subcoxae of legs I–III with 0,1/5–7,8–12/7–9,8–10 setae, respectively. Coxae of leg I with two front setae. Ventral tube with 4–5+4–5 latero-distal and 6–7 posterior setae (with four in distal transversal row), anteriorly without setae. Tenaculum with 4+4 teeth and one or two setae. Anterior furcal subcoxae with 11–16, posterior one with four setae. Anterior side of manubrium with 2+2 setae (rarely 2+3 or 1+2). Posterior side of manubrium with 4–5+4–5 latero-basal, two apical setae (ap), 2+2 setae in distal transversal row, pair of lateral setae present or absent (see the Discussion part), and 4–5(3–6)+4–5(3–6) in central part. Dens normally with 10–14 anterior setae (the whole range is 8–16). Posterior side of dens crenulated and with four setae: three (very rarely two) basal and one at the middle, no subapical setae. Mucro bidentate. Ratio of manubrium : dens : mucro = 3.1–4.9 : 2.4–4.3 : 1.

###### Remarks.

*Folsomia inoculata* is a rather peculiar species due to several characteristics. On Abd.V the differentiation of s-setae is unique: accp3-s is well-marked, tubular, and longer than three shortened and thin s-setae of “dorsal triplet” (shown in detail on fig.14 in Potapov and Greenslade 2010). The furca is of middle size, in an intermediate position between short-furcated ‘*tatarica*’ and long-furcated ‘*macrochaetosa*’ subgroups; posterior chaetotaxy of the dens is uncommon: seta at the middle present whereas subapical one normally absent (fig. XIV, 6 in Stach, 1947), the latter, although often small, is present in all other species of the ‘*inoculata*’ group. Appearance of the species is rather specific enabling its recognition under low magnification (Fig. 81).

Available vast material on this species shows a wide variation in several characters (chaetotaxy of manubrium and dens, shape of PAO, body length) which, however, are individual or population-dependent and does not indicate several species.

According to the original description, PAO is not constricted in *F. inoculata*, which was also shown in associated figures by Stach (1947, figs XIV, 7 and XIV, 8). This peculiarity was a reason for Niijima and Hasegawa (2011) to retain *F. ezoensis* Yosii, 1965 (described from Japan, PAO constricted) and *F. inoculata* Stach, 1947 (described from Poland, PAO not constricted) as two separate species. In our material, PAO is normally constricted in both western and eastern Palearctic (incl. Japan) while the character continuously varies depending on the specimen being, in fact, not constricted in an extreme variant (Fig. 83). A constricted PAO that is unlike the original description was also indicated for European populations by Martynova (1973), Schulz (1999), and Fjellberg (2007).

The modern detailed description of the species is given in Fjellberg (2007). Among other characteristics, a reduction of dorsal chaetom of manubrium was stressed, particularly lateral pair (l2) lost, only one pair of setae was shown in pr- and m-groups (fig. 23B–C in Fjellberg 2007). Such chaetotaxy was found by us only in specimens from Helgoland (NW Germany, coll. J. Schulz) (Fig. 86). Specimens from other localities normally have a pair of lateral setae and two or more pairs of setae at least in pr-group (Fig. 88). The presence of lateral setae l2 is not stable; few individuals missing them on both sides (sometimes asymmetrically) were recorded by us in the North Caucasus (Fig. 87), Germany, France, and Japan. Thus, a wide variation of chaetotaxy of posterior side of manubrium can be concluded for *F. inoculata*.

Size of the body ranges between 0.9 and 1.7. Specimens from eastern populations appears to be smaller than in western ones, but the whole variation is strongly overlapping (0.9–1.5 vs 1.1–1.7 mm, respectively).

The performed multivariate analysis of metric morphology did not reveal any irregularities, and noticeable differences between eastern and western populations were not detected (Fig. 89). Nevertheless, individuals of a particular population often resemble each other and this may be partly explained by the same phenological condition.

The species is facultatively parthenogenetic and its populations mostly consist of females. Males were seen by us only in four “central” localities: in Middle Ural mountains (upper flow of Pechora River), East Siberia (Podkamennaya Tunguska), Caucasus (Aibga Range), Turkey, and Kazakstan (West Altai).

###### Distribution and ecology.

**Figure 90.**
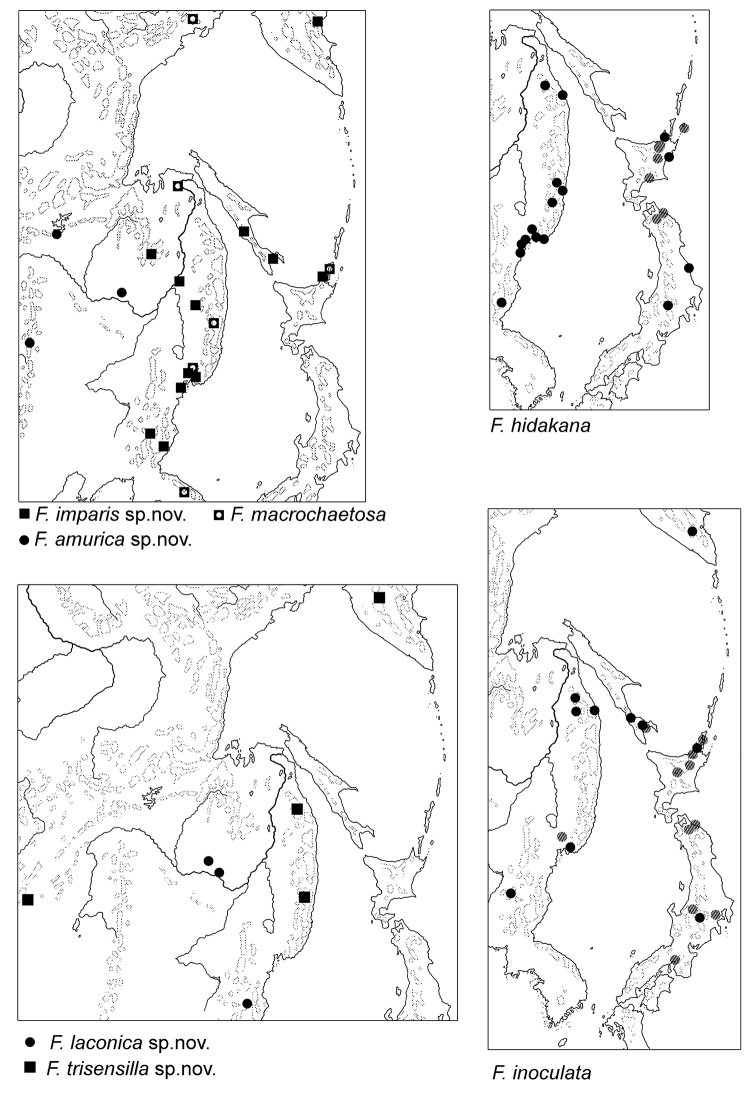
Records of species of the *Folsomia inoculata* group in eastern Asia. Striped circles–literature records (for references see the Distribution parts to associated species).

The species is widely distributed in the region (Fig. 90) and was listed in catalogues of Japanese Collembola (Yosii 1977; Furuno et al. 2000), often as its junior synonym *F. ezoensis*. Known from Osaka (Natuhara et al. 1994), Tokyo (Iwanami et al. 1980), Hokkaido (Yosii 1965, Suma 1997, Hishi et al. 2012), Shigayama (Tamura and Chiba 1977), Kyoto (Takeda 1995; Fujii and Takeda 2012), Aomori Pref. (Yamauchi and Suma 1999, 2009). For Russia, *F. inoculata* was recorded in Sakhalin (Kurcheva 1977; Solntseva and Molodova 1979), Ussuriyski Reserve and Kaimanovka (Kutyreva 1979, 1984, as *F. ezoensis*), Ussuriysk (Kutyreva 1988), and Kunashir Island (Potapov and Marusik 2000). In the revision of *Folsomia* of Russia the species was recorded in the Caucasus and the south of West Siberia (Martynova 1973).

Distributional range of *F. inoculata* appears to be restricted to the Holarctic. In the Palearctic we still have not seen specimens from the eastern areas of East Siberia, such as Buryat Republic and Amurskaya District, despite intensive collections in appropriate sites. Thus, all populations can probably be divided to ‘western’ and ‘eastern’ which are inseparable by morphology for the present. In Scandinavia and the westernmost part of Europe *F. inoculata* is very rare and appears to be an alien species (absent, for example, in the Iberian Peninsula). In the Nearctic, the species is also infrequent although it occurs at least on the Pacific coast of USA (Oregon, Cascade Range, coll. A. Smolis, our identification, new record). *Folsomia inoculata* does not occur in the Arctic; the northernmost record is north-east corner of Komi Republic (67.50°N, NE European part of Russia) (Babenko et al. 2017).

The species often occurs in forest litter while apparently preferring rotten wood where it can be very abundant. *Folsomia inoculata* is the most dendrophilous species of the ‘*inoculata*’ group and, very likely, in the genus, having associated shape of body and crushing mouth parts. The species is also sporadically recorded in specific sites enriched by organic matter.

### A key to the species of *Folsomia* of the ‘*inoculata*’ group

**Table:** 

1	Laterally positioned accp-3 s-seta much longer than three dorsal s-setae on Abd.V (Figs 7, 82). 8–16 setae on anterior side of dens	***inoculata* Stach, 1947** (Holarctic)
–	Accp-3 s-seta as long as or shorter than three dorsal s-setae on Abd.V (Figs 4–6). Anterior side of dens with 5–27 setae	**2**
2	More than eleven setae on anterior side of dens	**7**
–	Fewer than eleven setae on anterior side of dens	**3**
3	Medial s-setae in front of p-row on Th.II–III and Abd.I–II (Fig. 3)	**4**
–	Medial s-setae in p-row on Th.II–III and Abd.I–II (Fig. 1)	**5**
4	Abd.I without ms-setae (Fig. 71). Posterior side of manubrium with 3+3 latero-basal and 4+4 setae on main part (Fig. 73)	***calcarea* sp. n.** (south-east of European part of Russia)
–	Abd.I with ms-setae (as in Fig. 76). Posterior side of manubrium with 3+3 latero-basal and 6–7+6–7 setae on main part	***torpeda* Potapov, 2006** (north and east of European part of Russia, north-west of Siberia)
5	Medial s-setae in front of p-row on Abd.III (as in Fig. 71)	***tatarica* Martynova, 1964** (European part of Russia)
–	Medial s-setae in p-row on Abd.III (Fig. 77)	**6**
6	Dens with 5–7 anterior setae. Manubrium with one pair of setae on anterior side (Fig. 80)	***tubulata* sp. n.** (Yakutia)
–	Dens with 9–10 anterior setae. Manubrium with two pairs of setae on anterior side	***baida* Potapov, 2006** (north and north-east of European part of Russia)
7	Medial setae on ventral side of Th.III present (Fig. 9)	**8**
–	Medial setae on ventral side of Th.III absent (Fig. 10)	**16**
8	Medial s-setae on Th.III–Abd.II absent (s-formula: 42/11235)	***bashkira* Potapov, 2006** (Ural)
–	Medial s-setae on Th.III–Abd.II present (s-formula: 43/22235 or 33/22224)	**9**
9	Medial s-setae in front of p-row on Th.II–III (Fig. 2)	***brevisensilla* Potapov & Babenko, 2000** (North-East of Russia)
–	Medial s-setae within p-row on Th.II–III (Fig. 1)	**10**
10	One and three dorsal s-setae on Abd.IV and V, respectively (Fig. 6), corner s-setae on Th.II absent (Fig. 58): s-formula 33/22224. Ms-setae on Abd.I absent (Fig. 58)	**11**
–	Two and four dorsal s-setae on Abd.IV and V, respectively (Fig. 4), corner s-setae on Th.II present: s-formula 43/22235. Ms-setae on Abd.I present (Fig. 37)	**13**
11	Manubrium with 2+2 setae on anterior side and no more than 3+3 setae in central part of posterior side (Figs 67, 68)	***tertia* sp. n.** (Far East of Russia)
–	Manubrium with 3+3 or more setae on anterior side and more than 3+3 setae in central part of posterior side (Fig. 54)	**12**
12	Abd.V with p1-setae shorter than accp1 s-setae (Fig. 52). Three setae on basal part of posterior side of dens (Fig. 54)	***laconica* sp. n.** (Amurskaya District (Far East of Russia), North Korea)
–	Abd.V with p1-setae as long as accp1 s-setae (Fig. 53). Four setae on basal part of posterior side of dens (Figs 63–64)	***trisensilla* sp. n.** (East Siberia, Far East of Russia)
13	Setae on anterior side of ventral tube absent	**14**
–	Setae on anterior side of ventral tube present	***setifrontalis* Potapov & Marusik, 2000** (south of Far East of Russia)
14	Anterior side of manubrium with 2–3+2–3 setae (Fig. 48)	***macrochaetosa* Martynova, 1977** (Far East of Russia)
–	At whole, seven or more setae on anterior side of manubrium (Figs 33–35, 43–45)	**15**
15	Unpaired setae on anterior side of manubrium absent (Figs 33–35). Lateral accp3 s-seta clearly shorter and thicker than accp2 and accp1 on Abd V (Figs 30–31)	***amurica* sp. n.** (Amurskaya District (Far East of Russia), Inner Mongolia Province (North China))
–	Unpaired setae on anterior side of manubrium present (Figs 43–45). Lateral accp3 s-setae as long and as thick as accp2 and accp1 on Abd V (Fig. 40)	***imparis* sp. n.** (Japan, south of Far East of Russia)
16	Medial s-setae in front of p-row on Abd.I–III (Figs 3, 17). Body with short macrosetae	***breviseta* sp. n.** (North-East Asia)
–	Medial s-setae within p-row on Abd.I–III (Figs 2, 21, 22). Body with long macrosetae	***hidakana* Uchida & Tamura, 1968** (Japan, Korea, south of Far East of Russia)

## Supplementary Material

XML Treatment for Folsomia breviseta

XML Treatment for Folsomia hidakana

XML Treatment for Folsomia amurica

XML Treatment for Folsomia brevisensilla

XML Treatment for Folsomia imparis

XML Treatment for Folsomia macrochaetosa

XML Treatment for Folsomia setifrontalis

XML Treatment for Folsomia laconica

XML Treatment for Folsomia trisensilla

XML Treatment for Folsomia tertia

XML Treatment for Folsomia calcarea

XML Treatment for Folsomia tubulata

XML Treatment for Folsomia inoculata
